# Computational and Genetic Reduction of a Cell Cycle to Its Simplest, Primordial Components

**DOI:** 10.1371/journal.pbio.1001749

**Published:** 2013-12-31

**Authors:** Seán M. Murray, Gaël Panis, Coralie Fumeaux, Patrick H. Viollier, Martin Howard

**Affiliations:** 1 Computational and Systems Biology, John Innes Centre, Norwich Research Park, Norwich, United Kingdom; 2 Department of Microbiology & Molecular Medicine, Institute of Genetics & Genomics in Geneva (iGE3), Faculty of Medicine/CMU, University of Geneva, Geneva, Switzerland; National Cancer Institute, United States of America

## Abstract

What are the minimal requirements to sustain an asymmetric cell cycle? Here we use mathematical modelling and forward genetics to reduce an asymmetric cell cycle to its simplest, primordial components. In the *Alphaproteobacterium Caulobacter crescentus*, cell cycle progression is believed to be controlled by a cyclical genetic circuit comprising four essential master regulators. Unexpectedly, our *in silico* modelling predicted that one of these regulators, GcrA, is in fact dispensable. We confirmed this experimentally, finding that Δ*gcrA* cells are viable, but slow-growing and elongated, with the latter mostly due to an insufficiency of a key cell division protein. Furthermore, suppressor analysis showed that another cell cycle regulator, the methyltransferase CcrM, is similarly dispensable with simultaneous *gcrA*/*ccrM* disruption ameliorating the cytokinetic and growth defect of Δ*gcrA* cells. Within the *Alphaproteobacteria, gcrA* and *ccrM* are consistently present or absent together, rather than either gene being present alone, suggesting that *gcrA*/*ccrM* constitutes an independent, dispensable genetic module. Together our approaches unveil the essential elements of a primordial asymmetric cell cycle that should help illuminate more complex cell cycles.

## Abstract 2

Mathematical modelling and genetics in the bacterium *Caulobacter crescentus* identified redundancy in asymmetric cell cycle regulation through the dispensability of key transcription factor GcrA and methyltransferase CcrM, which together form a genetic module.

## Author Summary

Cell cycle regulation is remarkably complex and the fundamental principles difficult to understand, even in simple cells. The bacterium *Caulobacter crescentus* is a popular model organism to study cell cycle regulation due to the two different daughter cells resulting from cell division: a mobile “swarmer” cell and a “stalked” cell that adheres to surfaces. Here, we use mathematical modelling and genetic experiments to identify the core components of the asymmetric cell cycle of these bacteria. Using our mathematical model we predicted and confirmed experimentally that the transcription factor and cell cycle regulator, GcrA, hitherto thought to be essential, is in fact dispensable. We also identified another master regulator, the methyltransferase, CcrM as dispensable. Furthermore, simultaneous deletion of both GcrA and CcrM removes the severe cell division defects observed on either single deletion, returning cells to near wild-type morphology. We found that GcrA and CcrM constitute an independent, dispensable, genetic module that regulates transcription of cytokinetic proteins during the cell cycle. Phylogenetically, the module is conserved in *Alphaproteobacteria*, the class of *Caulobacter*, but is not present in the tree root of the class, suggesting that we have identified the primordial core of the asymmetric cell cycle regulatory circuit in the *Alphaproteobacteria*.

## Introduction

Replicatively asymmetric cell cycles exist where the two distinct daughter cells resulting from cell division have distinct abilities to replicate their DNA. This is the case for the many *Alphaproteobacteria* that reproduce by asymmetric binary fission (e.g., *Caulobacter* and *Brevundimonas* species) or budding (e.g., *Hyphomonas* and *Hyphomicrobium* species) to produce a motile swarmer cell from a nonmotile stalked mother cell (see [1] and references therein) [2],[3]. Swarmer cells do not replicate their DNA; they must first differentiate into stalked cells. During their motile juvenile phase, swarmer cells expend most of their energy on motility and little on growth [4],[5]. *Caulobacter crescentus* [6], a species that is ubiquitous in water, has for many years been used as a model organism for the study of development and the cell cycle. There are also examples of nonstalked bacteria that exhibit the same asymmetry in replication and motility (e.g., *Rhodopseudomonas palustris*) [7],[8]. Indeed, it has been proposed that morphological and functional asymmetry is much more widespread in the *Alphaproteobacteria* than previously thought [9]. This makes an understanding of asymmetric cell cycle regulation potentially even more relevant. However, the complexity of cell cycle control has made understanding the basic principles difficult. Here, we address this issue by using a minimal modelling approach to determine the core cell cycle regulatory circuit in *Caulobacter crescentus*.

**Figure 1:**
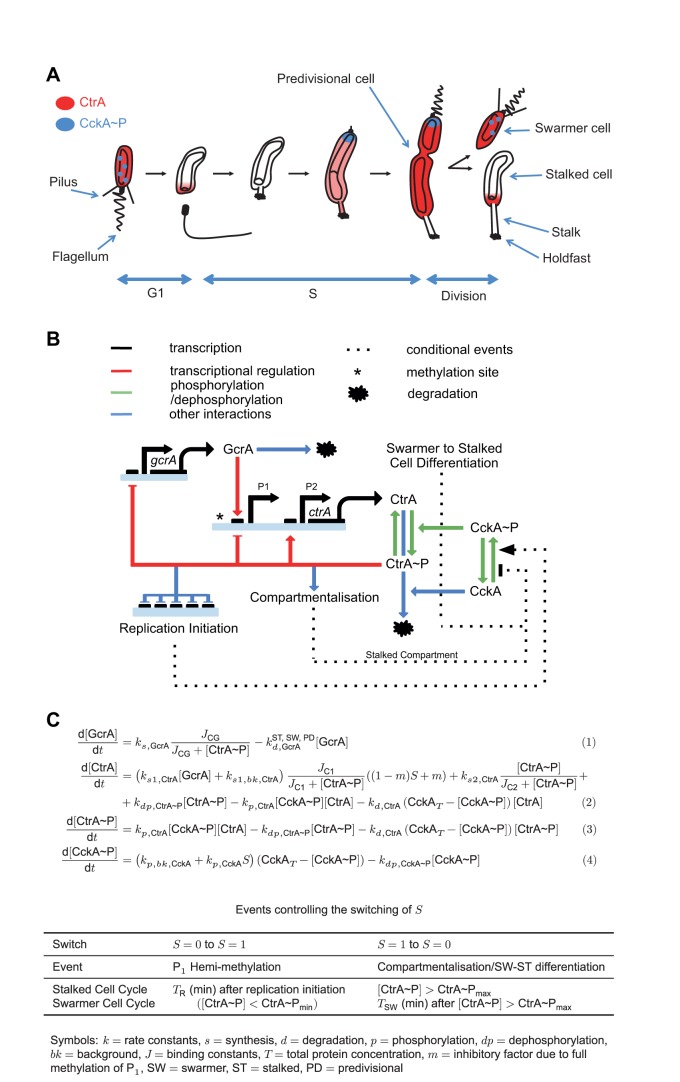
Minimal model of *Caulobacter crescentus* cell cycle. (A) Schematic of the cell cycle. Localization of (total) CtrA and phosphorylated CckA proteins indicated. (B) Circuit diagram of the mathematical model, reduced from the biological model shown in Figure 5B. Methylation and compartmentalisation are discrete events affecting *ctrA* transcription and activity of the CckA phosphorelay, respectively. (C) Mathematical description of model: ordinary differential equations and discrete events. See Text S1 for parameter values and justification.

Asymmetric division in *C. crescentus* yields a motile daughter swarmer (SW) cell and a sessile stalked (ST) cell. The ST cell immediately reinitiates replication, while the SW cell must differentiate into a ST cell before it can replicate and divide (Figure 1A). These replicative and morphological asymmetries are, in part, controlled by the essential master regulator CtrA through its ability, when activated by phosphorylation (CtrA∼P), to interact with DNA regulatory sequences in the origin of replication (*Cori*) and with many cell-cycle–regulated promoters [10],[11]. A second regulator of *Cori*, DnaA, ubiquitous in bacteria as an essential replication initiator, also targets many cell-cycle–regulated promoters [11],[12]. However, though DnaA levels are reduced in SW cells, they can support plasmid replication [13], indicating that it is likely not the regulator of replication asymmetry. This role is played by CtrA∼P: high levels inhibit replication in SW cells, whereas low levels in ST cells allow replication to proceed [14]. Instead, DnaA appears to dictate the underlying frequency of replication [15]. Localization of the activator and stabiliser of CtrA, the essential membrane-bound hybrid histidine kinase CckA [16], specifically at the future SW cell pole, ensures a high level of CtrA∼P in postdivisional SW cells and its removal in the ST compartment [17]. As *C. crescentus* regulates temporally both the abundance and activation of CtrA to control cell cycle progression [14], the cell cycle is very robust [18].

It has been proposed [19] that cell cycle progression in *C. crescentus* is controlled by a cyclical genetic circuit of four essential master cell cycle regulator proteins—DnaA, GcrA, CtrA, and CcrM—that are synthesised and degraded sequentially over the cell cycle. Here, we present a minimal mathematical modelling and experimental approach that challenges this assertion. Our model unexpectedly predicts that the “essential” cell cycle regulator GcrA is dispensable for core cell cycle progression. We experimentally test and verify this prediction. In addition, we experimentally uncover the dispensability of another cell cycle regulator, the methyltransferase CcrM, with simultaneous loss of the GcrA and CcrM module attenuating, rather than accentuating, cellular defects. Our conceptual approach resembles that applied to deciphering the minimal CDK control network in symmetrically dividing fission yeast [20], although here we study an inherently asymmetric cell cycle and also employ a mathematical modelling approach. We expect our results to hold in other *Alphaproteobacteria*, and our overall methodology should be useful in the dissection of more complex cell cycles.

## Results

### Mathematical Model

Asymmetric cell cycles are dictated by the spatiotemporally varying concentration of a regulatory protein whose presence inhibits DNA replication initiation in the nonreplicating offspring, whereas its absence in the “mother” cell allows replication (or *vice versa*). This asymmetry must begin at or before the time of compartmentalisation. Since replication factors are generally cytoplasmic and hence likely diffuse and equipartitioned, this suggests the existence of an additional, localised protein that controls the first's activation and/or stability. With the above regulatory module, a diffuse regulatory protein, and its localised activator/stabiliser, basic regulatory control of an asymmetric cell cycle should be possible. However, previous mathematical models [21]–[23] of asymmetric cell cycles (e.g., in *C. crescentus*) have been much more complex and have not made experimentally verified predictions. Here, we therefore develop a simple, but strongly predictive, mathematical model of an asymmetric cell cycle, as applied to *C. crescentus*, constructed to include only minimal regulatory elements.

The model incorporates GcrA, CckA, and CtrA, but not DnaA or CcrM (see justifications below). The cell cycle regulator GcrA regulates genes involving DNA replication, division, and polar development [24]. Its synthesis is promoted by DnaA (see below) and repressed by CtrA∼P. The *ctrA* gene has two promoters [25]: P_1_, activated by GcrA [24] but repressed by CtrA∼P and silenced by full DNA methylation [26], and P_2_, a stronger promoter, activated by CtrA∼P in a positive feedback loop. Halving of the P_1_ methylation state (hemi-methylation), with associated subsequent P_1_ activation, is due to movement of the DNA replication fork through the *ctrA* locus. This event is short in duration compared to other cell cycle timescales and is therefore modelled as a discrete event through the parameter S, which is switched from 0 to 1 at this time. We take the time at which CtrA∼P levels drop below a low threshold as synchronous with the assembly of the replication machinery at *Cori* and take P_1_ hemi-methylation to occur a fixed time later (the time required for replication initiation and subsequent movement of the replication fork past P_1_). The DNA methyltransferase CcrM, which has been reported to be essential for viability, remethylates the P_1_ promoter at the adenine within its GAnTC target site [27]. This remethylation (and as a result, silencing) occurs in late pre-divisional (PD) cells when P_1_ is already repressed and after *ccrM* has been activated by CtrA∼P. Therefore, for the model output, it is not relevant exactly when in late PD cells remethylation occurs, and we take it to be synchronous with compartmentalisation (for the ST compartment) or SW-ST differentiation (for the SW compartment), when the value of S is switched back to 0. The vital primary role of CcrM in resetting methylation-dependent promoters, as described above for the *ctrA* P_1_ promoter, is essentially discrete. Hence, although switching of P_1_ methylation is included, we do not explicitly include CcrM in our minimal model.

CckA initiates two phosphorelays leading to the phosphorylation (activation) and stabilisation of CtrA via a phosphotransferase, ChpT [28]. The active phosphorylated form, CckA∼P, is localised primarily to the pole opposite the stalk (Figure 1A) [29]. DivL, an essential noncanonical tyrosine kinase, activates, recruits, and co-localises with CckA [30],[31], events for which replication initiation is a prerequisite (handled in the model by a dependence on the parameter S) [32]. Upon compartmentalisation, the phosphotransfer from CckA is cut-off in the ST compartment. As a result, CtrA∼P is deactivated and removed from the ST cell progeny, while it remains stable and active in the SW cell (Figure 1A). This distinction is the fundamental origin of the asymmetry between the ST and SW cells. Diffusive exchange of cytoplasmic molecules is largely unimpeded up until the last moments of constriction [33]. Hence we model compartmentalization as a discrete event again through the discrete parameter S, which is switched from 1 to 0 at compartmentalisation but only in the ST compartment. This ST compartment-specific switch is the origin of the ST/SW asymmetry in the model. In the SW compartment, S is also eventually switched back to 0, but only at a much later time corresponding to the unknown signals initiating SW to ST differentiation. Furthermore, because high CtrA∼P levels activate the essential *ftsQA* cell division operon [34], we can take a high threshold level of CtrA∼P as a proxy for compartmentalisation. In summary, our model consists of the minimal regulatory module (CtrA and CckA) suggested above, the cell cycle regulator GcrA, the experimentally described *ctrA* promoter regulation, and three discrete cell cycle events (replication, compartmentalization, and SW–ST differentiation).

It has been proposed [19] that methylation of the *dnaA* promoter by the CcrM methyltransferase promotes *dnaA* transcription, thereby restarting the cascade of cell cycle regulators with a surge in DnaA synthesis. However, mutation of the putative methylation site in the *dnaA* promoter does not significantly alter promoter activity [35]. It is therefore unknown what leads to the burst in DnaA synthesis prior to replication initiation. It has recently been suggested that DnaA controls the initiation frequency rather than the acquisition of replication competence [15]. Due to this uncertainty and the focus of our minimal model on asymmetry, we do not explicitly include DnaA in our minimal model; we instead assume that DnaA levels rise as CtrA(∼P) levels drop and take a low threshold in CtrA∼P levels as being synchronous with replication initiation. This assumption is justified by the observation that CtrA and DnaA generally have alternating profiles—that is, when one is low, the other is high and *vice versa* [36], even under starvation conditions [37]—and CtrA proteolysis coincides with DnaA binding to *Cori* [38]. *In vitro*, CtrA binding displaces DnaA from *Cori*, indicating competitive binding [38]. The wiring diagram, equations, and parameters of the model are shown in Figure 1B,C. Further description and justification of the model are given in Text S1, and model SBML files are provided in Texts S2 and S3.

**Figure 2:**
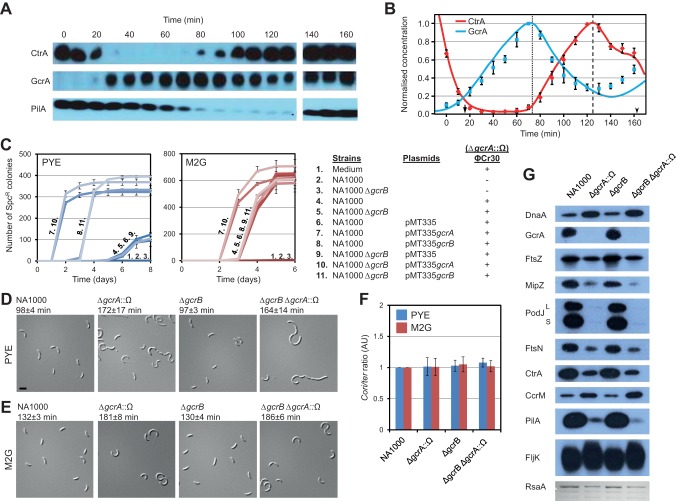
Δ*gcrA*::Ω mutant cells are viable but have growth and morphological defects. (A) Immunoblots showing GcrA, CtrA, and PilA (reporter for compartmentalization) steady-state levels for WT cells grown in M2G. (B) Simulated protein levels (solid lines) of GcrA and CtrA in WT cells, averaged over two compartments/cells where appropriate and incorporating imperfect cell cycle synchrony (see Text S1). Times of simulated events are indicated by an arrow (DNA replication initiation), an arrowhead (SW to ST differentiation of the SW daughter cell), a dotted line (*ctrA* P_1_ hemi-methylation), and a dashed line (compartmentalisation). CtrA and GcrA relative protein quantifications from immunoblots are the mean of three biological replicates; error bars are data ranges. Both datasets normalized to maximum ST/PD value. See Figure S1 for simulated profiles in ST and SW compartments and for *ctrA* promoter expression. (C) Time course of colony appearance following ΦCr30-mediated generalized transduction with Δ*gcrA*::Ω lysates from a Δ*gcrA*::Ω; *xylX::*P*_xyl_-gcrA* donor strain. Error bars are standard deviation from three biological replicates. Transductants scored on PYE (left panel, blue curves) or M2G (right panel, red curves) media supplemented with spectinomycin (30 µg/ml) and streptomycin (5 µg/ml) to select for Δ*gcrA*::Ω transduction. Table (right) shows conditions or strains used and number key for corresponding curves. Column (far right) shows whether Δ*gcrA*::Ω transducing lysate was added to cells or media. Total number of Spc^R^ clones obtained after transduction of cells containing the pMT335*gcrA* plasmid (7 and 10) reflects the efficiency of transduction of the Δ*gcrA*::Ω marker. Relative to this value, 33% in PYE and 96% in M2G of NA1000 transduced cells give Spc^R^ clones, confirming that NA1000 Δ*gcrA*::Ω colonies are not due to suppressors. (D, E) Differential interference contrast (DIC) micrographs of cells grown in PYE (D) or M2G (E). Scale bar represents 2 µm. Value above micrograph shows doubling time (with standard deviation from at least three biological replicates) for each strain. (F) Relative abundance of replication origin versus terminus (*Cori*/*ter* ratio) in WT and mutant cells grown in PYE (blue bars) or M2G (red bars). Ratios normalized to WT value. Triplicate measurements from two independent DNA extractions. Error bars are standard deviation. (G) Immunoblots showing steady-state levels of various proteins in WT and mutant cells in M2G. Lowest row shows levels of acid-extracted RsaA protein in M2G detected by Coomassie Brilliant Blue staining.

In order to select model parameter values, we used existing literature measurements (see Text S1). Out of 22 parameters, two are removed by normalisation, six are obtained from experiments, and 10 have constraints placed on their values by experiments. To constrain the model parameters further, we quantitated the relative amount of GcrA and CtrA during the same cell cycle at higher time resolution than previously (Figure 2A,B) by semiquantitative immunoblotting and fitted our model to the profiles. The small number of unknown parameters ensures that our model is constrained by the available data (see Text S1). The model recapitulated the known effects of various experimental perturbations in the methylation state of *ctrA* P_1_ or constitutive expression of GcrA (see Text S1 and Figure S2B). Comparisons between the theory and experiment are shown in Figure 2B and Figure S1, with good agreement.

### Mathematical Model Predicts and Experiments Confirm That GcrA Is Not Essential

Although the above model is simple and reproduces many features of the *C. crescentus* cell cycle, it is still more complex than our previous minimal two component module. In particular, we have included GcrA and intricate regulation of CtrA through two promoters. Consequently, we pursued a minimal two component model for *C. crescentus* by removing GcrA and its regulation of *ctrA* P_1_. Previously it was reported that GcrA-depleted cells die [24]. Accordingly, in previous models removal of GcrA led to failed cell cycle control. However, strikingly, our minimal *in silico* model predicted a functional cell cycle without GcrA, albeit with an extended period (Figure S2C). The model predicts that P_2_ promoter feedback, combined with the active (forward) CckA phosphorelay, is strong enough to raise CtrA levels without P_1_.

Prompted by these predictions, we revisited previous experimental approaches used to conclude that GcrA is indispensable. We inactivated *gcrA* by generalized transduction of a Δ*gcrA*::Ω allele (conferring spectinomycin resistance) into the NA1000 wild-type (WT) strain and found that colonies appeared after ∼6 d in rich medium (Figure 2C). By contrast, transduction into a WT strain harboring pMT335-*gcrA* (*gcrA* on multicopy plasmid pMT335) gave clones after ∼2 d. This 4-d growth delay may explain why *gcrA* was first described as essential in complex (PYE) medium [24]. On minimal (M2G) medium this delay is reduced to ∼1 d. However, the *CCNA_01269* (*CC_1211*, henceforth *gcrB*) gene of *C. crescentus* encodes an uncharacterized *gcrA* paralog with 44% sequence identity to *gcrA* (Figure S3). While ectopic expression of *gcrB* (from pMT335-*gcrB*) can partially compensate for the absence of *gcrA*, transduction of Δ*gcrA*::Ω into cells with an in-frame deletion in *gcrB* (Δ*gcrB*) gave similar results as transduction into WT cells (Figure 2C). PCR analysis (Figure S4A) and immunoblotting (Figure 2G) confirmed that transductants had exchanged the *gcrA* gene with the Δ*gcrA*::Ω allele. Importantly, the pleiotropic phenotypic defects of Δ*gcrA*::Ω cells (see below) were corrected by pMT335-*gcrA*, partially corrected by pMT335-*gcrB* but not by the empty vector (Figure S4B). Overall, these experiments confirm a key prediction of our model that GcrA is inessential for cell cycle progression.

### Consequences of GcrA Deletion

We first focused on the cell doubling time during exponential growth, as determined by the optical density of a liquid culture. This time is dependent on the type of growth medium used. We found that the doubling time of Δ*gcrA*::Ω cells was 75% longer than for the WT in PYE, and 40% longer in M2G, qualitatively consistent with the model (Figure 2D,E). The cells also exhibited a lengthened lag phase (unpublished data). However, Δ*gcrA*::Ω cells have an origin-to-terminus ratio similar to WT cells, demonstrating that this lengthened doubling time is not due to a defect in DNA replication initiation (Figure 2F, see below). Consistent with these results, fluorescence-activated cell sorting (FACS, Figure S5) revealed an increase in cell length (Figure S5A) and in chromosome number (Figure S5B) in Δ*gcrA*::Ω versus WT cells. The increase in chromosome number scales with the increase in cell length, which arises from perturbed cytokinesis (Figure 2D,E, see below). Immunoblotting showed a reduction in levels of the MipZ division regulator and the essential late cell division protein FtsN (Figure 2G). Finally, the difference in cell length in Δ*gcrA*::Ω versus WT cells was attenuated in M2G (176% of WT) compared to PYE (264% of WT), consistent with the respective increases in doubling time described above.

We also observed that Δ*gcrA*::Ω cells do not undergo the cell-cycle–regulated switch in buoyancy that is exploited to separate swarmer and stalked cells (Figure S4B) and are poorly motile on soft (0.3% PYE) agar (Figure S4C) and in broth (unpublished data). Additionally, Δ*gcrA*::Ω cells are resistant to the S-layer–specific bacteriophage ΦCr30 and the pilus-specific bacteriophage ΦCbK (Figure S4B) consistent with reduced levels of the pilin subunit PilA, the polarity factor PodJ (both required for pilus assembly), and of the S-layer subunit RsaA in Δ*gcrA*::Ω versus WT cells (Figure 2G).

The abundance of CtrA, DnaA, and CcrM was also altered in Δ*gcrA*::Ω cells: while CtrA was diminished (as expected from our model), the abundances of CcrM and DnaA were elevated (Figure 2G). Importantly, immunoblotting revealed identical defects on protein abundance seen in Δ*gcrA*::Ω already after 5 h of GcrA depletion using the xylose-inducible promoter (P*_xyl_*). These defects were still present 24 h after depletion but were reversed following re-instatement of *gcrA* expression for 16 h (Figure S6A,C).

### Lack of FtsN Is the Major Cause of Defects in Δ*gcrA*::Ω Cells

**Figure 3:**
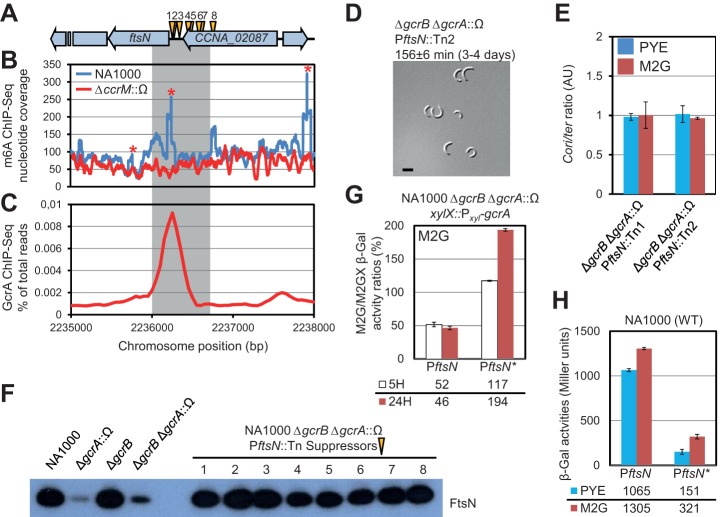
Δ*gcrA*::Ω cells suffer from insufficiency in FtsN. (A) Position of eight *himar1* transposon (Tn) insertions (yellow arrowheads) that ameliorate slow growth of Δ*gcrA*::Ω mutant cells on PYE. (B) Trace of N6-methyladenosine (m6A) marked DNA at *ftsN* locus of WT cells from ChIP-Seq experiment performed with antibodies to m6A. Figure reports number of times given nucleotide position sequenced from chromatin precipitates. Stars show predicted positions of GAnTC methylation sites at *ftsN* locus. (C) Occupancy of GcrA across *ftsN* locus as determined by ChIP-Seq using polyclonal antibodies to GcrA. Occupancy expressed as percentage of total reads. Chromosome coordinates below chart in (C) match scheme shown in (A). In (A–C) grey-shaded region denotes *ftsN* promoter fragment (P*_ftsN_*) used in promoter probe experiments shown in (G, H). (D) DIC image of Δ*gcrB* Δ*gcrA*::Ω cells harbouring P*_ftsN_*::Tn2 insertion [depicted in (A)] grown in PYE. Scale bar represents 2 µm. Values above micrograph indicate doubling time (with standard deviation from three biological replicates) and, in brackets, the number of days after which the majority of colonies are visible. (E) Relative *Cori*/*ter* ratio in Δ*gcrB* Δ*gcrA*::Ω cells harbouring either P*_ftsN_*::Tn1 or P*_ftsN_*::Tn2 insertion. Cells grown in PYE (blue bars) or M2G (red bars). Ratios normalized to WT value. Triplicate measurements from two independent DNA extractions. Error bars are standard deviation. (F) Immunoblots showing steady-state levels of FtsN in WT, *gcrA* mutants, and derivatives harbouring different Tn insertions at *ftsN* locus in PYE. (G) P*_ftsN_*- or P_ftsN__*_-*lacZ* (GAnTC mutant) activity after depletion of GcrA for 5 h (white) or 24 h (red) in Δ*gcrB* Δ*gcrA*::Ω *xylX::*P*_xyl_-gcrA* cells grown in M2GX (M2G containing 0.3% xylose) or depleted by washing and cultivation in M2G. At 24 h of GcrA depletion, the absolute levels of β-galactosidase are 637±8 Miller units for P*_ftsN_* and 608±6 Miller units for P_ftsN*_. (H) β-galactosidase activity of P*_ftsN_*- or P_ftsN__*_-*lacZ* fusions in NA1000 cells grown in PYE (blue) or M2G (red), values below histograms. In (G, H) data are from four independent experiments; error bars are standard deviation.

To test if one or a combination of these abnormalities impairs growth of Δ*gcrA*::Ω cells, we screened for Δ*gcrB* Δ*gcrA*::Ω cells mutagenized with an *himar1* transposon (Tn) that form colonies faster than the parent. Backcrossing and mapping identified nine distinct Tn insertions, eight of which were in either the 5′ end of *ftsN* or in the 3′ end of the upstream gene, *CCNA_02087* (*CC_2008*), that reads in the same direction as *ftsN* (Figure 3A). These eight P*_ftsN_*::Tn insertions attenuate the growth and division defect of Δ*gcrA*::Ω cells as demonstrated by DIC imaging (cf., Figure 3D with Figure 2D) and by FACS (Figure S5A,B). Moreover, immunoblotting revealed that these insertions restore FtsN to near WT levels (Figure 3F), presumably because of an outwardly facing promoter of the Tn directing *ftsN* transcription. As before, the origin-to-terminus ratios were similar to the WT (Figure 3E). We also confirmed that transduction of Δ*gcrA*::Ω into WT cells expressing FtsN from P*_van_* on pMT335 (pMT335-*ftsN*) or from P*_xyl_* at the chromosomal *xylX* locus (Δ*ftsN xylX::*P*_xyl_-ftsN*) gave colonies after ∼3 d on rich medium (unpublished data), compared to ∼6 d for the WT expressing FtsN from the endogenous promoter. Consistent with functional interaction of *ftsN* and *gcrA*, accumulation of *ftsN* mRNA was shown before to be GcrA-dependent [24]. Using a *lacZ*-based transcriptional reporter plasmid, we confirmed that P*_ftsN_* indeed requires GcrA for full activity. After 5 h and 24 h of GcrA depletion, P*_ftsN_*-*lacZ* is reduced to 52% and 46% of WT activity, respectively (Figure 3G). Chromatin immunoprecipitation using antibodies to GcrA followed by deep-sequencing (ChIP-Seq) revealed that GcrA binds the *ftsN* promoter (P*_ftsN_*) *in vivo* (Figure 3C), suggesting that *ftsN* is a direct target of GcrA. In sum, activation of P*_ftsN_* by GcrA is critical for efficient growth and division, and reduced FtsN levels cause growth defects in Δ*gcrA*::Ω cells.

### *ccrM* Is Dispensable for Viability

**Figure 4:**
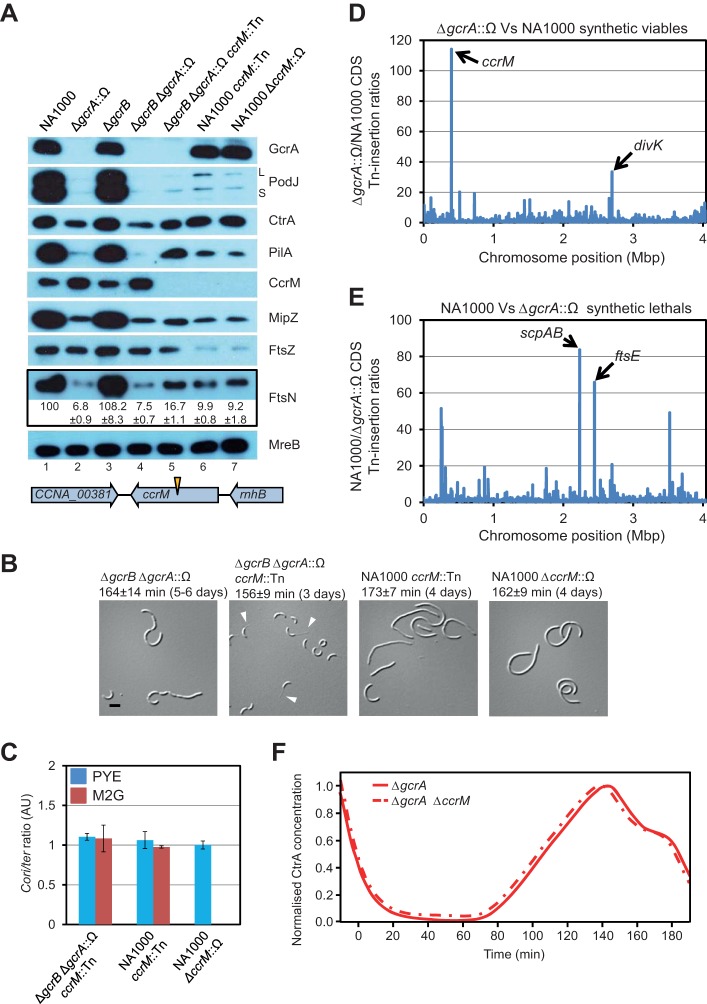
Dispensability and genetic interactions of *gcrA*-*ccrM* regulatory module. (A) Immunoblots showing abundance of various proteins in WT and mutant strains in PYE. FtsN relative protein quantifications were normalized to the NA1000 sample value and represent the average and standard deviation of three independent experiments. Schematic shows Tn position in *ccrM*::Tn allele. (B) Representative DIC micrographs of *gcrA* and *ccrM* mutant cells grown in PYE. Leftmost figure reproduced from Figure 2D. Scale bar represents 2 µm. Arrowheads indicate stalks. Values above micrograph show doubling time for each strain (with standard deviation from at least three biological replicates) and, in brackets, the number of days after which the majority of colonies are visible. (C) Relative *Cori*/*ter* ratio in mutant strains grown in PYE (blue bars) or M2G (red bars). Ratios normalized to WT value. Triplicate measurements from two independent DNA extractions. Error bars are standard deviation. (D) Tn insertion bias in coding sequences (CDS) of Δ*gcrA*::Ω cells relative to WT cells as determined by Tn-seq. Abscissa shows position as function of genome position, and ordinate gives insertion ratio. Peaks show CDSs with the highest number of Tn insertions. Noncoding sequences are not included (see Figure S7). (E) Inverse ratio shown compared to ratio in (D), with peaks indicating CDSs receiving fewest insertions in Δ*gcrA*::Ω cells relative to WT cells. (F) Simulated protein levels of CtrA in Δ*gcrA* (solid line) and Δ*gcrA* Δ*ccrM* (dashed line) averaged as in Figure 2B. Both curves normalised to maximum PD value.

Surprisingly, one Tn of the nine insertions was found in the middle of the *ccrM* gene (*ccrM*::Tn, Figure 4A). We performed complementation experiments and found that WT cells expressing *ccrM* from P*_van_* on pMT335 (pMT335-RBS-*ccrM*) formed colonies on PYE ∼3 d after transduction of *ccrM*::Tn compared to ∼5 d for WT cells harbouring the empty vector (unpublished data). We also found that *ccrM*::Tn could be transduced into WT cells on PYE and that genomic DNA extracted from the *ccrM*::Tn mutant is susceptible to cleavage by the methylation-sensitive restriction enzyme *Hin*f1, as is the case for genomic DNA extracted from Δ*ccrM*::Ω cells (Figure S7A) [39]. Immunoblotting with antibodies to CcrM provided further confirmation that *ccrM*::Tn is a null allele (Figure 4A). Therefore, *ccrM*, like *gcrA*, is dispensable for viability, consistent with the recent report by Gonzalez and Collier [40]. However, *ccrM*::Tn colonies take 4 d to form on PYE, similar to Δ*ccrM*::Ω, and present a lengthened doubling time (Figure 4B). This slow growth rate may explain why *ccrM* was previously reported to be essential [27]. As before, neither the Δ*gcrA*::Ω mutation nor the *ccrM*::Tn mutation affect the origin-to-terminus ratio compared to the WT (Figure 4C).

### Tn-Insertions in *ccrM* Are Greatly Overrepresented in Δ*gcrA*::Ω Cells as Compared to WT

To quantitatively evaluate the relationship between *gcrA* and *ccrM*, we determined the relative frequency of Tn insertions in WT and Δ*gcrA*::Ω mutant cells by Tn-Seq following *himar1* Tn mutagenesis. Tn insertions were hugely overrepresented along the *ccrM* coding sequence for Δ*gcrA*::Ω cells compared to the WT (Figure S7B), being ∼115 times more abundant than the average of insertions over other coding sequences (Figure 4D). By contrast, Tn insertions in *scpAB* and *ftsE*, both with promoters bound by GcrA *in vivo* based on ChIP-Seq (Table S1), are underrepresented in Δ*gcrA*::Ω compared to the WT (Figure 4E). Insertions in the region upstream of *ftsN* were also found to be greatly overrepresented in Δ*gcrA*::Ω cells compared to the WT (Figure S7C) and were even more frequent than in the *ccrM* sequence (Figure S7B) consistent with the number and location of the nine insertions found in the Tn suppressor screen (Figures 3A and 4A). We also observed an increased bias in insertions in the *gcrB* promoter region (Table S3), confirming the previously observed partial complementation of Δ*gcrA*::Ω.

### *ccrM*::Tn Ameliorates the Defects of Δ*gcrB* Δ*gcrA*::Ω Cells

Returning to the *ccrM*::Tn insertion found in the screen, we discovered that it greatly improves the cytokinetic defect of Δ*gcrB* Δ*gcrA*::Ω cells (Figure 4B). This was quantified with FACS analyses (Figure S5), which showed a substantial reduction in cell length and chromosome number, and with a variance much closer to WT than that of Δ*gcrB* Δ*gcrA*::Ω P*_ftsN_*::Tn2 cells. We also observed a reduced lag phase (unpublished data), though there was little or no improvement in the doubling time. The presence of stalked cells in DIC images indicted that morphological asymmetry is at least partially maintained in Δ*gcrB* Δ*gcrA*::Ω *ccrM*::Tn cells (Figure 4B). To investigate this further, we examined the localisation pattern of the stalked pole-specific marker SpmX [41] in Δ*gcrA*::Ω *ccrM*::Tn cells and found a unipolar focus of SpmX-mCherry at the same site as the stalk (Figure S8A), confirming that morphological asymmetry is maintained. Consistent with elevated steady-state levels of PilA (Figure 4A), we also observed that the resistance of Δ*gcrA*::Ω cells to ΦCr30 and ΦCbK is diminished by the *ccrM*::Tn mutation (Figure S4B). Lastly, we addressed replicative asymmetry by studying the localisation pattern of the centromere-binding protein ParB, which binds to the *parS* site near the origin of replication. Localisation of GFP-ParB [42] revealed an uneven number of foci in Δ*gcrA*::Ω cells, consistent with the presence of replicative asymmetry (Figure S8B). Most importantly, time-lapse imaging of GFP-ParB in Δ*gcrA*::Ω *ccrM*::Tn cells showed asymmetric duplication and segregation of GFP-ParB (Figure S8C), strongly suggesting that replicative asymmetry is maintained in these cells.

### *ftsN* Is Regulated by CcrM Methylation

We next explored the regulatory basis for the Tn insertion enrichment. The results of the suppressor screen and Tn-seq suggest that lack of FtsN is a significant limiting growth factor in Δ*gcrB* Δ*gcrA*::Ω cells. Therefore, we reasoned that the recovery due to the *ccrM*::Tn insertion might, at least partially, be mediated through *ftsN*. Accordingly we noted that P*_ftsN_* also harbors a GAnTC methylation site. We found in ChIP-seq experiments that a polyclonal antibody to N6-methyladenosine (m6A) [39] precipitated P*_ftsN_* efficiently from WT but not from Δ*ccrM*::Ω cells (Figure 3B), indicating that P*_ftsN_* carries a CcrM-dependent m6A mark overlapping the GcrA target site. To evaluate the importance of this methylation site, we mutated the GAnTC site to GTnTC and found in *lacZ*–promoter probe assays (Figure 3H) that the mutant (P_ftsN*_) promoter fires only at 14% of the WT rate in PYE (25% in M2G), consistent with reduced FtsN levels when *ccrM* is disrupted (Figure 4A, lanes 1 and 6). Moreover, P_ftsN*_ activity doubles by 24 h after depletion of GcrA from Δ*gcrB* cells (Figure 3G), consistent with increased FtsN levels in the Δ*gcrB* Δ*gcrA*::Ω *ccrM::*Tn mutant compared to *ccrM::*Tn (Figure 4A, lanes 5 and 6), while, as noted earlier, the activity of WT P*_ftsN_* responds in the opposite fashion (Figure 3G). However, the activities, in absolute units, of P*_ftsN_* and P_ftsN*_ 24 h after depletion of GcrA in the Δ*gcrB* background are very similar (637±8 and 608±6 Miller units, respectively). This would suggest that the methylation state of P*_ftsN_* has an effect only in the presence of GcrA and that therefore the recovery in FtsN levels observed in Δ*gcrB* Δ*gcrA*::Ω *ccrM::*Tn cells compared to Δ*gcrB* Δ*gcrA*::Ω (Figure 4A, lanes 4 and 5) is probably not mediated through the *ftsN* promoter (assuming the mutation does not have unwanted side effects on the firing of the core promoter), at least on a low-copy *lacZ*-reporter plasmid. Because methylation is transient, the net effect on P*_ftsN_* may result in the same measured activity as P_ftsN*_, but the timing of methylation could be important or the results may be skewed due to a low level of GcrA expression being maintained from P*_xyl_* even under repressive conditions that is absent in the Δ*gcrB* Δ*gcrA*::Ω *ccrM::*Tn mutant. However, taken together, these data suggest that *ftsN* is positively regulated by GcrA in the presence of methylation but negatively regulated in its absence.

FtsN abundance in Δ*gcrB* Δ*gcrA*::Ω *ccrM::*Tn is still considerably lower than in WT or Δ*gcrB* Δ*gcrA*::Ω P*_ftsN_*::Tn cells, even though the aberrant division of Δ*gcrB* Δ*gcrA*::Ω cells is largely repaired. The improved variance in cell length and chromosome number and reduced lag in growth of Δ*gcrB* Δ*gcrA*::Ω cells carrying the *ccrM::*Tn versus the P*_ftsN_*::Tn mutation (see above) is likely due to the pleiotropic nature of the mutation, elevating the expression of many division genes to some extent. This is consistent with the somewhat raised steady state levels of CtrA and MipZ in Δ*gcrB* Δ*gcrA*::Ω *ccrM::*Tn cells compared to Δ*gcrB* Δ*gcrA*::Ω (Figure 4A, lanes 4 and 5).

Because *ccrM* mutants also exhibit a reduction in FtsN abundance compared to WT cells (Figure 4A), we tested if transduction of Δ*ccrM*::Ω into Δ*ftsN xylX::*P*_xyl_-ftsN* yielded colonies on PYE (with 0.3% xylose) similar to the Δ*gcrA*::Ω mutation. Transduced Δ*ftsN xylX::*P*_xyl_-ftsN* colonies appeared after ∼4 d, whereas transduced WT colonies expressing FtsN from the endogenous promoter only appeared after 5–6 d (unpublished data). Thus, the growth defect of *ccrM* mutants can be improved by expression of extra FtsN (in addition to extra FtsZ [40]).

### The Mathematical Model Provides an Explanation for the Lack of Recovery in Doubling Time

Disruption of *ccrM* ameliorates the cytokinetic and morphological defects of Δ*gcrB* Δ*gcrA*::Ω cells but not the doubling time (Figures 2D and 4B). Returning to our model, we asked if these experimental findings could be recapitulated. We found in our simulations that maintaining *ctrA* P_1_ in the unmethylated state (mimicking the loss of *ccrM*) in Δ*gcrA* cells caused only a slight change in cell cycle timing compared to the loss of GcrA alone (Figure 4F), with a ∼3% decrease in the swarmer cell cycle period, consistent with the experimentally observed trend (Figure 4B). Without methylation to suppress early activation, our model suggests that basal *ctrA* P_1_ transcription in Δ*gcrA* cells results in premature synthesis of CtrA and so has a negative effect on cell cycle progression, which cancels the positive effect during CtrA re-accumulation. The same neutral effect on doubling time was also seen in a WT background (Figure S2B) consistent with previous results (Text S1) [26]. Our model therefore provides a possible explanation for why the decrease in cell doubling time is small, even though Δ*gcrB* Δ*gcrA*::Ω *ccrM::*Tn cells otherwise show substantial phenotypic recovery versus Δ*gcrB* Δ*gcrA*::Ω cells.

## Discussion

**Figure 5:**
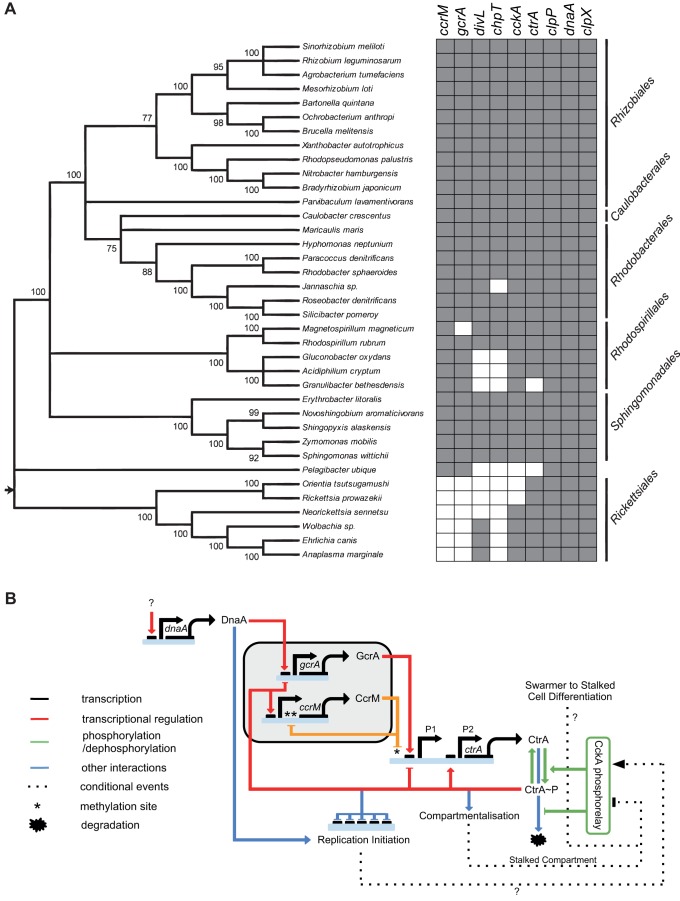
Coconservation of *gcrA*/*ccrM* suggests a primordial cell cycle regulatory circuit. (A) *gcrA* and *ccrM* are generally coconserved in the *Alphaproteobacteria* (adapted from Figure 2 and Table S2 of [43]). One species of each genus analysed is shown (37 out of a total of 65 genomes). (B) Current biological model of cell cycle regulation in *Caulobacter crescentus*, with the dispensable GcrA/CcrM module highlighted. Question marks indicate elements of the circuit that have yet to be fully elucidated.

Our combined minimal modelling and forward genetics approach in *C. crescentus* has unexpectedly uncovered *gcrA* and *ccrM* as a dispensable genetic module. Strikingly, the core *C. crescentus* asymmetric cell cycle network can therefore function without two of the four “master” regulators, revealing a high level of robustness. Though both *gcrA* and *ccrM* are highly conserved, it is much more common for both to be present or absent in the *alphabacterial* lineages rather than either gene being present alone (Figure 5A) [43], which supports our findings. Furthermore, *gcrA* and *ccrM* are not present in the tree root of the *Alphaproteobacteria*, whereas *ctrA* and *cckA* are [43], suggesting that our minimal model describes the cell cycle of the primordial *Alphaproteobacterium*. Within our minimal cell cycle network, we find that asymmetry can be controlled with just two fundamental components: an inhibitor of DNA replication initiation (CtrA) and a localised activator that controls the former's cell-type–specific activation (CckA). In Figure 5B we present a schematic of the current biological model of cell cycle regulation in *C. crescentus* with the dispensable GcrA/CcrM module highlighted. Several elements have yet to be fully understood (indicated by question marks): What triggers the pulse in DnaA concentration at the beginning of the cycle? Is this mediated by the Lon protease [44] and/or transcriptional control of *dnaA* [19]? How are CckA localisation and activation dependent on replication initiation? What are the mechanisms underlying SW cell differentiation?

The co-evolution of GcrA and CcrM and our results on *ftsN* regulation are consistent with the recent discovery that promoter binding and transcriptional activation by GcrA is methylation-dependent [39]. Connecting transcriptional activation to chromosome position and cell cycle timing (via the hemi-methylation caused by the passing of the replication fork) potentially contributes greatly to cell cycle robustness, an important property especially for oligotrophic bacteria. From the point of view of the circuit shown in Figure 5B the GcrA/CcrM module could contribute to robustness in two ways: (1) Methylation-regulated transcription of *ctrA* P_1_ controls the timing and prevents the early accumulation of CtrA, which would hinder cell cycle progression; (2) GcrA provides an additional connection between DnaA and CtrA, resulting in more reliable accumulation and activation of CtrA after DNA replication initiation, in addition to the link between DNA replication initiation and CckA localisation/activation.

The potential role of the GcrA/CcrM module in the robustness of the timing of cell cycle events may also explain why its absence is primarily observed in obligate endosymbionts and pathogens, as are found in the *Rickettsiales*. These bacteria have a long generation time as an adaption to the slower growth of the host cell. For example, *Rickettsia prowazekii* has a doubling time of about 10 h. This is likely reflected in a more relaxed coordination of cell cycle events with replication fork progression, coordination for which the GcrA/CcrM module is of great importance. Thus, without strong selection, it is perhaps not surprising that this module was lost during the evolution of this branch.

The success of our approach in reducing an asymmetric cell cycle to its simplest, primordial components illustrates the potential to define the core regulation of sophisticated cell cycles using this strategy. This methodology would be especially useful for eukaryotic cell cycles. The complexity of their regulatory networks has made understanding the basic principles involved a challenging task. Mathematical models of eukaryotic cell cycles, such as those of fission and budding yeast [45]–[47], have made limited progress in simplifying the regulatory networks or identifying essential, core components. Indeed, progress on identifying redundancy in the cell cycle circuitry has been led by experiments rather than by modelling [20],[48]. We therefore expect that our minimal modelling approach could play an important role in dissecting these more complex cell cycles.

## Materials and Methods

### Growth Conditions

*Caulobacter crescentus* NA1000 [49] and derivatives were cultivated at 30°C in peptone yeast extract (PYE) rich medium or in M2 minimal salts plus 0.2% glucose (M2G) supplemented by 0.4% liquid PYE [50]. *Escherichia coli* S17-1 [51] and EC100D (Epicentre Technologies, Madison, WI) were cultivated at 37°C in Luria Broth (LB) rich medium. We added 1.5% agar into M2G or PYE plates, and motility was assayed on PYE plates containing 0.3% agar. Antibiotic concentrations used for *C. crescentus* include kanamycin (solid, 20 µg/ml; liquid, 5 µg/ml), tetracycline (1 µg/ml), spectinomycin (liquid, 25 µg/ml), spectinomycin/streptomycin (solid, 30 and 5 µg/ml, respectively), gentamycin (1 µg/ml), and nalidixic acid (20 µg/ml). When needed, D-xylose or sucrose was added at 0.3% final concentration.

Swarmer cell isolation, electroporation, biparental mating, and bacteriophage φCr30-mediated generalized transduction were performed as described in [50],[52],[53].

### Bacterial Strains, Plasmids, and Oligonucleotides

Bacterial strains, plasmids, and oligonucleotides used in this study are listed and described in tables below.

### Plasmid Constructions

#### pNPTS138*-ΔgcrB-KO*

The plasmid construct used for *gcrB* (*CCNA_01269* or *CC_1211*) deletion was made by PCR amplification of two fragments. The first, a 633 bp fragment flanked by an *Eco*R1 site at the 5′ end and a *Bam*HI at the 3′ end (amplified using primers delgcrB_1-EcoRI and delgcrB_1-BamHI), encompasses the upstream region of *gcrB* and extends to 15 bp downstream of the predicted start codon. The second fragment, flanked by a *Bam*HI site at the 5′ end and a *Hind*III site at the 3′ end (amplified using primers delgcrB_2-BamHI and delgcrB_2-HindIII), harbors the last 6 bp of the *gcrB* coding sequence and extends 629 bp downstream of the gene. These two fragments were first digested with appropriate restriction enzymes and then triple ligated into pNTPS138 (M.R.K. Alley, unpublished) that had been previously restricted with *Eco*RI and *Hin*dIII.

#### pNPTS138*-ΔrsaA*

The plasmid construct used for *rsaA* (*CCNA_01059* or *CC_1007*) disruption was made by PCR amplification of a 691 bp fragment that overlaps the *rsaA* coding sequence (+1,500 to +2,191 relative to the TTG start codon, amplified using primers delrsaA-EcoRI and delrsaA-HindIII). This fragment is flanked by a *Hin*dIII site at the 5′ end and an *Eco*RI at the 3′ end, and then cloned into corresponding sites of the pNTPS138 after hydrolysis with the appropriate restriction enzymes.

#### pMT335*gcrA*

The *gcrA* coding sequence was PCR amplified from the Δ*gcrA*::Ω P*xylX*::*gcrA* strain [24] using the PxylX and gcrA-EcoRI primers. This fragment was digested with *Nde*I/*Eco*RI and cloned into pMT335 [54].

#### pMT33*5gcrB*

The *gcrB* (*CCNA_01269* or *CC_1211*) coding sequence was PCR amplified from NA1000 using gcrB-NdeI and gcrB-EcoRI and cloned into pMT335 using *Nde*I-*Eco*RI.

#### pMT335*-RBS-ccrM*

The *ccrM* coding sequence (*CCNA_00382* or *CC_0378*) was amplified using ccrM-RBS-EcoRI and ccrM-XbaI and cloned into pMT335 using *Eco*RI and *Xba*I. The ccrM-RBS-EcoRI primer encoded an optimized RBS to optimize CcrM translation.

#### pMT335*ftsN*

The *ftsN* (*CCNA_02086* or *CC_2007*) coding sequence was PCR amplified from NA1000 using ftsN-NdeI and ftsN-EcoRI and cloned into pMT335 using *Nde*I and *Eco*RI.

#### p*lacZ*290-*pftsN*

The *ftsN* promoter region (−469 to +228 relative to the ATG) was PCR-amplified using pftsN-EcoRI and pftsN-XbaI primers using NA1000 chromosomal DNA as a template. This fragment was digested by appropriate enzymes and cloned into a *Eco*RI*-Xba*I-digested p*lacZ*290 promoter probe vector.

#### p*lacZ*290-pftsN*

Same as previous, using the same pftsN-EcoRI and pftsN-XbaI primers and synthesized pftsN* DNA fragment (DNA2.0 Inc, Menlo Park, CA) as a template where the A position (−52 relative to the ATG) of the GATTC site is replaced by a T.

### Strain Constructions

#### NA1000 Δ*gcrB*

pNPTS138-Δ*gcrB*-KO was first introduced into NA1000 (WT) by intergeneric conjugation and then plated on PYE harboring kanamycin (to select for recombinants) and nalidixic acid to counter select *E. coli* donor cells [50]. A single homologous recombination event at the *CCNA_01269* locus of kanamycin-resistant colonies was verified by PCR. The resulting strain was grown to stationary phase in PYE medium lacking kanamycin. Cells were then plated on PYE supplemented with 3% sucrose and incubated at 30°C. Single colonies were picked and transferred in parallel onto plain PYE plates and PYE plates containing kanamycin. Kanamycin-sensitive cells, which had lost the integrated plasmid due to a second recombination event, leaving a deleted version of *gcrB* behind (Δ*gcrB*), were then identified for disruption of the *gcrB* locus by PCR.

#### NA1000 Δ*rsaA*::pNPTS138

As above, the pNPTS138-Δ*rsaA* plasmid was introduced into NA1000 by intergeneric conjugation. Kanamycin-resistant ex-conjugants having undergone a single homologous recombination event were isolated and the integration was verified by PCR.

#### NA1000 Δ*gcrB* Δ*gcrA*::Ω *xylX*::P*xyl*-*gcrA*

The *xylX*::P*xyl*-*gcrA* (Kan^R^) construction from LS3707 [24] was first transduced into NA1000 Δ*gcrB* using φCr30. Next, the Δ*gcrA*::Ω (Spc^R^) allele was then transduced into NA1000 Δ*gcrB xylX*::P*xyl*-*gcrA* and plated on solid PYE containing spectinomycin/streptomycin antibiotics and 0.3% xylose.

#### NA1000 *ccrM*::Tn

The *ccrM*::Tn (399278^+^) insertion (Kan^R^) from LT419 was transduced into NA1000 using φCr30 and plated on solid PYE containing kanamycin.

#### NA1000 *spmX*-*mCherry* Δ*gcrA*::Ω *ccrM*::Tn

The Δ*gcrA*::Ω (Spc^R^) allele from LS3707 and *ccrM*::Tn (399278^+^) insertion (Kan^R^) from LT419 were successively transduced using φCr30 into NA1000 *spmX*-*mCherry* [41].

#### NA1000 *egfp*-*parB* Δ*gcrA*::Ω *ccrM*::Tn

The Δ*gcrA*::Ω (Spc^R^) allele from LS3707 and *ccrM*::Tn (399278^+^) insertion (Kan^R^) from LT419 were successively transduced using φCr30 into NA1000 *egfp*-*parB* [42].

### φCr30 Transduction of the ΔgcrA Mutation

Δ*gcrA*::Ω (Spc^R^) transducing phage stock is a φCr30 lysate of LS3707 [24]. Overnight cultures of NA1000 and NA1000 *ΔgcrB* strains harboring or not pMT335, pMT335*gcrA*, or pMT335*gcrB* plasmids were first washed with fresh liquid medium (PYE or M2G) and resuspended at a concentration of 10^9^ cfu/ml. We infected 0.5 ml of cells with 50 µL of φCr30 phage stock (∼10^10^ pfu/ml), incubated them for 2 h at room temperature, and then plated them on solid PYE or M2G containing spectinomycin/streptomycin antibiotics. Plates were incubated at 30°C, and visible colonies were counted each day. Experimental values represent the average of three biological replicates. Addition of 50 mM vanillate to the plates, to further induce GcrA or GcrB synthesis from pMT335 plasmids (that harbor *gcrA* or *gcrB* under the control of the vanillate-inducible promoter, P*van* [54]), did not change the transduction values, presumably due to leaky expression from P*van*. Integration of the Δ*gcrA*::Ω construction was checked by PCR and confirmed by immunoblot analysis. PCR was done using pro-gcrA and gcrA-EcoRI primers that allow amplification of the *gcrA* gene only in strains that do not carry the Δ*gcrA*::Ω allele. Strains harboring the pMT335*gcrA* complementation plasmid served as a positive control of φCr30 transduction efficiency. The fact that the ratio of NA1000+pMT335 versus NA1000+pMT335*gcrA* colonies counted on PYE or M2G plates are 0.38 and 1.05, respectively, indicates that spontaneous suppressors are not frequent. In support of this, genome sequencing of several Δ*gcrA*::Ω derivatives failed to reveal suppressor mutations.

### Cori/ter Ratio Determination

Cells were grown to exponential phase in PYE or M2G medium and then harvested. Chromosomal DNA was extracted using Ready-Lyse Lysozyme solution (Epicentre Biotechnologies) and DNAzol Reagent (Invitrogen) and then precipitated in 100% ethanol. The DNA pellet was washed 3 times with 70% ethanol and incubated at room temperature in 8 mM NaOH solution for 4 h. HEPES 1 M (pH 7) was added to neutralize the pH. Cori-fwd/Cori-rev and Ter-fwd/Ter-rev primers were used to amplify ∼170 bp DNA close to the origin (*Cori*) or close to the terminus (*ter*), respectively, of the *Caulobacter crescentus* chromosome [55]. Real-time PCR was performed using the Step-One Real-Time PCR system (Applied Biosystems) at different DNA dilutions (5 µL), with 12.5 µL of SYBR green PCR master mix (Quanta Biosciences), 0.5 µL of each primer (10 µM), and 6.5 µL of water per reaction. PCR assay parameters were one cycle at 95°C for 5 min followed by 40 cycles at 95°C for 15 s, 55°C for 20 s, and 60°C for 15 s. Dilutions of NA1000 extracted DNA were used to generate *Cori* and *ter* standard curves, involving all *Cori*/*ter* ratios being normalized to the WT value. Average values are from triplicate measurements from two independent DNA extractions.

### β-Galactosidase Assays

β-Galactosidase assays were performed at 30°C as described previously [52],[56]. We lysed 50 µL of washed cells at OD_660 nm_ = 0.1–0.6 with chloroform and mixed them with 750 µL of Z buffer (60 mM Na_2_HPO_4_, 40 mM NaH_2_PO_4_, 10 mM KCl, and 1 mM MgSO_4_ heptahydrate). We added 200 µL of ONPG (4 mg/ml o-nitrophenyl-β-D-galactopyranoside in 0.1 M KPO_4_ pH 7.0), and the reaction was timed. When a medium-yellow color developed, the reaction was stopped with 400 µL of 1 M Na_2_CO_3_. The OD_420 nm_ of the supernatant was determined and the units were calculated with the equation: U = (OD_420 nm_ * 1000)/(OD_660 nm_ * time (in min) * volume of culture (in ml)). For GcrA depletion experiments in M2G using strain NA1000 Δ*gcrB* Δ*gcrA*::Ω *xylX*::P*xyl*-*gcrA*, M2G supplemented with 0.3% xylose overnight cultures were harvested and washed 3 times with M2 minimal salt solution, and then restarted in appropriate M2G or M2GX medium for 5 or 24 h at 30°C. For the 24 h time point, culture dilutions were done to maintain cells in exponential growth phase. Experimental values represent the averages of four independent experiments.

### GcrA Depletion Experiment

For GcrA depletion experiments, overnight cultures of strain NA1000 Δ*gcrB* Δ*gcrA*::Ω *xylX*::P*xyl*-*gcrA* grown in M2G supplemented with 0.3% xylose were harvested and washed 3 times with M2 minimal salt solution, and then resuspended in M2G (GcrA depletion) or M2GX (GcrA expression) medium for 2, 5, or 24 h at 30°C (Figure S6A). Then, the 24 h M2G culture was supplemented with 0.3% xylose and incubated with the 24 h M2GX culture for an additional 16 h at 30°C. For the 24 h and 40 h time points, culture dilutions were done to maintain cells in exponential growth throughout the experiment.

### Immunoblot Analysis

Protein samples were separated by SDS-PAGE and blotted on PVDF (polyvinylidenfluoride) membranes (Merck Millipore). Membranes were blocked for 1 h with phosphate buffered saline (PBS), 0.05% Tween 20, and 5% dry milk and then incubated for an additional 1 h with the primary antibodies diluted in PBS, 0.05% Tween 20, 5% dry milk. The different antisera were used at the following dilutions: anti-DnaA (1∶20,000) [38], anti-GcrA (1∶5,000) [24], anti-FtsZ (1∶20,000) [57], anti-MipZ (1∶5,000) [42], anti-PodJ (NTD) (1∶10,000) [58], anti-FtsN (1∶10,000) [59], anti-CtrA (1∶10,000) [14], anti-CcrM (1∶10,000) [27], anti-PilA (1∶10,000) [60], and anti-FljK (1∶20,000) [61]. The membranes were washed 4 times for 5 min in PBS and incubated for 1 h with the secondary antibody diluted in PBS, 0.05% Tween 20, and 5% dry milk. The membranes were finally washed again 4 times for 5 min in PBS and revealed with Immobilon Western Blotting Chemoluminescence HRP substrate (Merck Millipore) and Super RX-film (Fujifilm). For CtrA and GcrA relative protein quantifications during the cell cycle (Figure 2B), membranes were scanned using the LAS-4000 digital imaging system (Fujifilm) and analyzed with the Multi Gauge V3.0 software. Each protein quantification value was normalized to the OD_660 nm_. Both datasets were normalized to their maximum value. Data represent the averages of three independent synchrony experiments. Each membrane was used for only one antibody detection to avoid any cross-reaction. For FtsN relative protein quantifications (of unsynchronized cultures), datasets were normalized to the NA1000 sample value and represent the average of three independent experiments.

### RsaA Acid Extraction

Cell cultures of 5 ml were grown to exponential phase in M2G medium and then harvested. Cells were washed twice with 5 ml of 100 mM HEPES (pH 7.2) and then resuspended in 200 µL of 100 mM HEPES (pH 2). After 10 min of incubation at room temperature, cells were pelleted and removed. The supernatant pH was neutralized by adding 3 µL of 5N NaOH solution. Samples were separated on 7.5% acrylamide SDS-PAGE gel followed by Coomassie Blue staining.

### Microscopy

PYE or M2G cultivated cells in exponential growth phase were immobilized using a thin layer of 1% agarose. For time-lapse experiments, LT494 cells in exponential growth phase were immobilized using a thin layer of PYE supplemented with 1% agarose. Fluorescence and contrast microscopy images were taken with an Alpha Plan-Apochromatic 100×/1.46 DIC(UV) VIS-IR oil objective on an Axio Imager M2 microscope (Zeiss) with 405 and 488 nm lasers (Visitron Systems GmbH, Puchheim, Germany) and a Photometrics Evolve camera (Photometrics) controlled through Metamorph V7.5 (Universal Imaging). Images were processed using Metamorph V7.5.

### FACS

Cells in exponential growth phase (OD_660 nm_ = 0.3–0.6), cultivated in PYE or M2G, were fixed in ice cold 70% Ethanol solution. Fixed cells were resuspended in FACS staining buffer pH 7.2 (10 mM Tris-HCl, 1 mM EDTA, 50 mM NaCitrate, 0.01% TritonX-100) and then treated with RNase A (Roche) at 0.1 mg/ml for 30 min at room temperature. Cells were stained in FACS staining buffer containing 0.5 µM of SYTOX Green nucleic acid stain solution (Invitrogen) and then analyzed using a BD Accuri C6 flow cytometer instrument (BD Biosciences, San Jose, CA). Flow cytometry data were acquired and analyzed using the CFlow Plus V1.0.264.15 software (Accuri Cytometers Inc.). We analyzed 20,000 cells from each biological sample. The forward scattering (FSC-A) and Green fluorescence (FL1-A) parameters were used to estimate cell sizes and cell chromosome contents, respectively. Relative chromosome number was directly estimated from the FL1-A value of NA1000 cells treated with 20 µg/ml Rifampicin for 3 h at 30°C. Rifampicin treatment of cells blocks the initiation of chromosomal replication, but allows ongoing rounds of replication to finish.

### Cell Generation Time Determination

Cell growth in PYE or M2G medium was done in an incubator at 30°C under agitation (190 rpm) and monitored at OD_660 nm_. Generation time values were extracted from the curves using the Doubling Time application (http://www.doubling-time.com). Values represent the averages of at least three independent clones.

### Chromatin Immunoprecipitation Coupled to Deep Sequencing (ChIP-Seq)

Midlog phase cells cultivated in PYE were cross-linked in 10 mM sodium phosphate (pH 7.6) and 1% formaldehyde at room temperature for 10 min and thereafter on ice for 30 min, then washed three times in PBS, and lysed in a Ready-Lyse lysozyme solution (Epicentre Biotechnologies, Madison, WI) according to the manufacturer's instructions. Lysates were sonicated (Sonifier Cell Disruptor *B*-*30*) (Branson Sonic Power Co., www.bransonic.com) on ice using 10 bursts of 20 s at output level 4.5 to shear DNA fragments to an average length of 0.3–0.5 kbp and cleared by centrifugation at 14,000 rpm for 2 min at 4°C. Lysates were then diluted to 1 ml using ChIP buffer (0.01% SDS, 1.1% Triton X-100, 1.2 mM EDTA, 16.7 mM Tris-HCl (pH 8.1), 167 mM NaCl plus protease inhibitors (Roche, www.roche.com)) and precleared with 80 µl of protein-A agarose (Roche, www.roche.com) and 100 µg BSA. Polyclonal antibodies to GcrA [24] were added to the remains of the supernatant (1∶1,000 dilution), incubated overnight at 4°C with 80 µl of protein-A agarose beads pre-saturated with BSA, washed once with low salt buffer (0.1% SDS, 1% Triton X-100, 2 mM EDTA, 20 mM Tris-HCl (pH 8.1), 150 mM NaCl), high salt buffer (0.1% SDS, 1% Triton X-100, 2 mM EDTA, 20 mM Tris-HCl (pH 8.1), 500 mM NaCl), and LiCl buffer (0.25 M LiCl, 1% NP-40, 1% sodium deoxycholate, 1 mM EDTA, 10 mM Tris-HCl (pH 8.1)) and twice with TE buffer (10 mM Tris-HCl (pH 8.1) and 1 mM EDTA). The protein–DNA complexes were eluted in 500 µl freshly prepared elution buffer (1% SDS, 0.1 M NaHCO_3_), supplemented with NaCl to a final concentration of 300 mM and incubated overnight at 65°C to reverse the crosslinks. The samples were treated with 2 µg of Proteinase K for 2 h at 45°C in 40 mM EDTA and 40 mM Tris-HCl (pH 6.5). DNA was extracted using phenol∶chloroform∶isoamyl alcohol (25∶24∶1), ethanol-precipitated using 20 µg of glycogen as a carrier, and resuspended in 100 µl of water.

HiSeq 2000 runs of barcoded ChIP-Seq libraries yielded several million reads that were mapped to *Caulobacter crescentus* NA1000 (NC_011916) according to the ELAND alignment algorithm (services provided by Fasteris SA, Switzerland). The standard genomic position format files (BAM) were imported into SeqMonk (Braham http://www.bioinformatics.babraham.ac.uk/projects/seqmonk/, version 0.21.0) to build sequence read profiles. The initial quantification of the sequencing data was done in SeqMonk to allow the comparison of different conditions and to isolate regions of interest. To this end, the genome was subdivided into 50 bp probes, and for every probe an associated value was calculated, a value that derives from the pattern of reads that occurs within the probe region used for the quantitation using the Red Count Quantitation option. To discern between background signal (modeled with a Poisson or negative binomial distribution) and candidate peaks, we calculated the ratio of reads per probe as a function of the total number of reads. The overall average read count (for all probes) plus twice the standard deviation was used to establish the lower cut-off that separates the background from candidate peaks. Analyzed data are provided in Table S1 with selected peak values highlighted in yellow. Figure 3C focuses this analysis on the *ftsN* and *CCNA_02087* region (2,235,000 to 2,238,000 bp on the *Caulobacter crescentus* genome). Figure 3B used global m6A ChIP-Seq analysis data obtained in the laboratory [39].

### Transposon Suppressor Screen and Mapping

An overnight Δ*gcrB* Δ*gcrA*::Ω cell culture was grown in PYE and mutagenized with a *himar1* transposon (Tn) [62]. To create the *himar1* strain collection, transposition was induced by mobilizing the *himar1* transposon (Kan*^R^*) from plasmid pHPV414 in *Escherichia coli* S17-1 into the NA1000 Δ*gcrB* Δ*gcrA*::Ω strain and selecting for kanamycin-nalidixic acid-resistant *Caulobacter* clones that form colonies faster than the parent at 30°C (∼5/6 d) on PYE. Nine distinct Tn clones appearing after ∼3 d were selected and already showed, under the microscope, less filamentous defects than the parent strain. Using φCr30-mediated generalized transduction, the Tn insertions were backcrossed in NA1000 Δ*gcrB* followed by transduction of the Δ*gcrA*::Ω strain to verify the phenotypes. Chromosomal DNA of the nine selected suppressors was extracted and partially digested for 4 min with *Hin*P1 restriction. Digested DNA was recircularized by T4 DNA ligase (Roche) treatment and then electroporated in *E. coli* EC100D *pir+* 116 (Epicentre Biotechnologies). Plasmids of kanamycin-resistant clones were extracted and mapped using the himar-Seq2 primer that allowed the sequencing of the DNA region on the *Caulobacter crescentus* chromosome adjacent to the Tn insertion region.

### Transposon Suppressor Screen Coupled to Deep Sequencing (Tn-Seq)

Tn collections of >100,000 kanamycin-nalidixic acid-resistant clones were collected for NA1000 (WT) and Δ*gcrA*::Ω strains, with the same protocol as previously described [62]. For each collection, all Tn clones were mixed and chromosomal DNA extracted. This DNA was used to generate a barcoded ChIP-Seq library and submitted to Illumina HiSeq 2000 sequencing. Tn insertion-specific reads (50 bp long) were sequenced using the himar-Tnseq primer and yielded several million reads that were mapped to *Caulobacter crescentus* NA1000 (NC_011916) [63] according to the ELAND alignment algorithm (Johann Mignolet, Methods, manuscript in preparation). The Tn insertion coordinate format files (BED) were generated and imported into SeqMonk V0.21.0 for analyses. Table S3 is an excel table and includes NA1000-TnSeq and Δ*gcrA*::Ω-TnSeq data, where values are assigned for each Tn integration position on the *Caulobacter crescentus* chromosome. Both datasets were normalized to the total number of reads of the largest dataset. This file was used to generate Figure S7B and Figure S7C that are respectively focused on the *ccrM* and *ftsN* regions. Table S2 is also an excel table and includes Δ*gcrA*::Ω-TnSeq/NA1000-TnSeq ratio analyses. To this end, the annotated genome was subdivided into coding sequence (CDS) probes (precluding the analysis of noncoding sequences such as promoter regions), and for every probe, an associated Tn insertion value was calculated. Both datasets were normalized to the total number of reads of the largest dataset. An average value of all CDS-Tn insertions normalized to the gene size was calculated, and 1% of this normalized value was used to correct each CDS-Tn insertion value. This correction prevents, during ratio calculations, a CDS-Tn insertion value of 0 and excludes also from the analyses CDS that do not share sufficient statistical Tn insertions. This file was used to generate Figure 4D and 4E.

## Supporting Information

Figure S1 **Simulated GcrA/CtrA profiles.** (A, B) Simulated concentrations of GcrA and (total) CtrA and tracked into the SW (A) and ST (B) compartments at the single cell level. Concentrations have the same normalisation factor as in Figure 2B. (C) Simulated CtrA synthesis from the *ctrA* P_1_ and P_2_ promoters (solid lines) is in good qualitative agreement with published plasmid-monitored expression profiles (points and dashed lines) [26]. Curves are normalised to peak *ctrA* P_2_ expression. (A–C) Times of simulated events are indicated by arrows (DNA replication initiation), an arrowhead (SW to ST differentiation of the SW daughter cell), dotted lines (*ctrA* P_1_ hemi-methylation), and dashed lines (compartmentalisation).
(EPS)

Figure S2 **Predicted GcrA/CtrA profiles.** (A) Simulated concentrations from Figure 2B reproduced for comparison. (B) Simulated GcrA and CtrA concentrations of synchronised cells with *ctrA* P_1_ promoter maintained in its hemi-methylated state. The SW cell cycle period is very similar to the WT consistent with [26]. (C) Simulated CtrA concentration of synchronised Δ*gcrA* cells. The SW cell cycle period is 13% longer than the WT. (A–C) Times of simulated events are indicated as in Figure S1.
(EPS)

Figure S3 **Identification of** ***gcrA*** **paralog.** The *gcrA* coding sequence (Holtzendorff et al., 2004) [24] was blasted (Sbjct) against the *Caulobacter crescentus* genome (NC_011916.1) using the NCBI online blastx application (http://blast.ncbi.nlm.nih.gov/). The typical result of this query is presented. This analysis allows identification of the *CCNA_01269* (Query) as a putative GcrA protein paralog, sharing 44% sequence identity and henceforth denoted *gcrB*.
(EPS)

Figure S4 **Confirmation and phage sensitivity of Δ** ***gcrA*** **::Ω cells.** (A) Verification of the Δ*gcrA*::Ω deletion after transduction into NA1000 (WT) cells. Colonies appearing after 5–6 d on PYE (upper panel) or M2G (lower panel) plates (supplemented with spectinomycin 30 µg/ml and streptomycin 5 µg/ml) were screened by PCR using primers Pro-gcrA and gcrA-EcoRI, revealing that the majority of the 17 tested colonies had exchanged endogenous *gcrA* with Δ*gcrA*::Ω. This primer pair amplifies a 594 bp fragment when endogenous *gcrA* is present, while only a primer dimer band is seen for the Δ*gcrA*::Ω background. As positive (C+) and negative (C−) controls, PCR amplifications were also carried out with NA1000 and Δ*gcrA*::Ω, *xylX::*P*_xyl_-gcrA* cells. (B) Sensitivity of WT and mutant cells to the S-layer specific phage φCr30 and the pilus-specific phage φCbK. Serial dilutions of φCr30 and φCbK were spotted on lawns of cells embedded in the top-agar on PYE plates. Spot tests on WT and *gcrA* mutants carrying various plasmids (as indicated) are shown in the frame. The first column shows controls with WT, flagellin (Δ*fljx6*), pilus (Δ*pilA*), and *rsaA* mutants (Δ*rsaA*). The schematics to the right of the spot test represent test tubes showing the buoyancy of the strain. WT cells give two buoyancies (high and low). The mutants are affected in this property. The thickness of the bands reflects the number of cells obtained with a given buoyancy. (C) Motility assays of WT and various mutants on PYE soft (0.3%) agar plates. Complementation experiments of WT and *gcrA* mutants carrying various plasmids (as indicated) are shown in the frame. The first column shows negative control of swarming using a flagellin (Δ*fljx6*) mutant.
(EPS)

Figure S5 **Relative cell size and chromosome number distributions analysed by FACS for NA1000 and various mutants.** (A) Relative cell size distributions in NA1000 and various mutants cultivated to exponential phase in PYE or M2G medium were analysed by flow cytometry. Forward scattering values (FSC-A) were used to estimate the cell size. A total of 20,000 cells were analysed for each population, and a density heat map was used to represent the cell population distribution as a function of the FSC-A parameter. Median values are indicated (white lines) and normalized to the median value of NA1000 cultivated in PYE. (B) Same FACS samples acquired in (A) were analysed using the green fluorescence parameter (FL1-A) to estimate the relative chromosome number distribution. Additional NA1000 sample treated with Rifampicin (+Rif) was used to obtain a correlation between the FL1-A parameter and the relative chromosome number. A total of 20,000 cells were analysed for each population, and a density heat map was used to display the cell population distribution as a function of the FL1-A parameter. Median values are indicated (white lines).
(EPS)

Figure S6 **Depletion experiment supports the finding that GcrA is not essential.** (A) Scheme summarizes the experimental approach used to prepare GcrA depleted samples in (C). NA1000 Δ*gcrB* Δ*gcrA*::Ω *xylX*::P*xyl*-*gcrA* grown in M2G supplemented with 0.3% xylose were harvested and washed 3 times with M2 minimal salt solution, and then resuspended in M2G (GcrA depletion) or M2GX (GcrA expression) medium for 2, 5, or 24 h at 30°C. Then, the 24 h M2G culture was supplemented with 0.3% xylose (G+X) and incubated with the 24 h M2GX culture (X) for an additional 16 h at 30°C. For the 24 h and 40 h time points, culture dilutions were done to maintain cells in exponential growth throughout the experiment. (B) Immunoblots showing steady-state levels of various proteins in WT and mutant cells in M2G (reproduced from Figure 2G). (C) Immunoblots showing steady-state levels of various proteins after 2, 5, or 24 h of GcrA depletion in M2G. Red rectangle highlights that 5 h of GcrA depletion are sufficient to reconstruct Δ*gcrA*::Ω protein steady-state profiles shown in (B). After 24 h of GcrA depletion in M2G, culture was supplemented with 0.3% xylose (G+X) and incubated with the 24 h M2GX culture (X) for an additional 16 h at 30°C to obtain, respectively, X and G+X 40 h samples. Blue rectangle indicates that 16 h of GcrA reinduction restores WT protein steady-state profile, confirming that prolonged depletion of GcrA phenocopies the effect of the Δ*gcrB* Δ*gcrA*::Ω strain and that WT protein levels can be restored by reinstatement of GcrA expression. This argues against the possibility that suppressive mutations accumulate in the Δ*gcrB* Δ*gcrA*::Ω strain under these conditions.
(EPS)

Figure S7 **Tn-insertion bias at the** ***ccrM*** **and** ***ftsN*** **loci and confirmation that the** ***ccrM*** **::Tn insertion is a null allele.** (A) Chromosomal DNA of NA1000 and of some Δ*gcrA*::Ω and/or *ccrM*::Tn variants were purified and submitted (+) or not (−) to *Hin*f1 restriction analysis. *Hin*f1 can only cleave unmethylated GAnTC sites. The fact that the *ccrM*::Tn (as with the Δ*ccrM*::Ω) extracted DNA is sensitive to restriction confirms that the *ccrM*::Tn mutation is a null allele. (B, C) Tn-insertion bias of Δ*gcrA*::Ω (blue) and NA1000 (red) strains determined by Tn-seq at the *ccrM* (B) and the *ftsN* (C) loci. Abscissa shows position as function of genome position, and ordinate gives Tn-insertion value. This Tn-Seq approach confirmed the Tn-suppressor screen, Tn-integration accumulating specifically all along the *ccrM* coding sequence and the *ftsN* promoter region in Δ*gcrA*::Ω strain compared to the WT.
(EPS)

Figure S8 **The Δ** ***gcrA*** **::Ω** ***ccrM*** **::Tn strain retains morphological and replicative asymmetry.** (A) Fluorescence and DIC micrographs of NA1000, Δ*gcrA*::Ω, and Δ*gcrA*::Ω *ccrM*::Tn, *spmX*-*mCherry* cells after growth in PYE. In all three strains, when a stalk structure is visible on the DIC micrograph, the stalked-pole-specific marker SpmX reveals unipolar SpmX-mCherry localization at this site, confirming that elongated Δ*gcrA*::Ω strain and Δ*gcrA*::Ω *ccrM*::Tn double mutant retain morphological asymmetry. Arrowheads indicate stalks. (B) Fluorescence and DIC micrographs of NA1000, Δ*gcrA*::Ω, and Δ*gcrA*::Ω *ccrM*::Tn, *egfp*-*parB* cells after growth in PYE. Localization of the centromere binding protein GFP-ParB revealed an uneven number of foci in *gcrA* elongated cells, consistent with replicative asymmetry still being intact. Δ*gcrA*::Ω *ccrM*::Tn double mutant is equivalent to the WT strain. (C) Fluorescence and DIC time lapse imaging of Δ*gcrA*::Ω *ccrM*::Tn *egfp*-parB predivisional cell in PYE. First duplication of GFP-ParB foci in the new stalked compartment argues that Δ*gcrA*::Ω *ccrM*::Tn double mutant retains replicative asymmetry.
(EPS)

Table S1 **GcrA ChIP-Seq analysis on the NA1000 chromosome.**
(XLSX)

Table S2 **Δ** ***gcrA*** **::Ω/NA1000 CDS Tn-insertion ratios.**
(XLSX)

Table S3 **Global Tn-insertion location values of NA1000 and Δ** ***gcrA*** **::Ω datasets.**
(XLSX)

Text S1 **Supporting experimental procedures.**
(PDF)

Text S2 **SBML file describing the SW cell cycle model.**
(XML)

Text S3 **SBML file describing the ST cell cycle model.**
(XML)

## Abbreviations

PD: Pre-divisional
ST: Stalked
SW: Swarmer
