# Supplementary material for: Computational and Genetic Reduction of a Cell Cycle to Its Simplest, Primordial Components
Source: PLoS Biol. 2013 Dec 31;11(12):e1001749. doi: 10.1371/journal.pbio.1001749 (PMC3885167; doi:10.1371/journal.pbio.1001749)
Supplement: Text S1 — Supporting experimental procedures. (PDF) [file pbio.1001749.s012.pdf]

Supporting Experimental Procedures

Strains

	Characteristics	Reference/source
Escherichia coli		
S17	RP4,Tc::Mu Km::Tn7	[1]
EC100D	*F- mcrA Δ(mrr-hsdRMS-mcrBC) Φ80dlacZΔM15 ΔlacX74 recA1 endA1 araD139 Δ(ara, leu)7697 galU galK λ- rpsL (StrR) nupG*	Epicentre
Caulobacter crescentus		
NA1000	Synchronizable derivative of wild-type strain CB15	[2]
LS3707	NA1000 Δ*gcrA*::Ω *xylX*::P*xyl-gcrA*	[3]
LT406	NA1000 Δ*gcrB*	This work
LT407	NA1000 Δ*gcrA*::Ω	This work
LT408	NA1000 Δ*gcrB* Δ*gcrA*::Ω	This work
LT409	NA1000 Δ*gcrB xylX*::P*xyl-gcrA*	This work
LT410	NA1000 Δ*gcrB* Δ*gcrA*::Ω *xylX*::P*xyl-gcrA*	This work
LT411	NA1000 Δ*gcrB* Δ*gcrA*::Ω p*ftsN*::Tn1 (2236255$^+$)	This work
LT412	NA1000 Δ*gcrB* Δ*gcrA*::Ω p*ftsN*::Tn2 (2236257$^+$)	This work
LT413	NA1000 Δ*gcrB* Δ*gcrA*::Ω p*ftsN*::Tn3 (2236315$^+$)	This work
LT414	NA1000 Δ*gcrB* Δ*gcrA*::Ω p*ftsN*::Tn4 (2236429$^+$)	This work
LT415	NA1000 Δ*gcrB* Δ*gcrA*::Ω p*ftsN*::Tn5 (2236431$^+$)	This work
LT416	NA1000 Δ*gcrB* Δ*gcrA*::Ω p*ftsN*::Tn6 (2236590$^+$)	This work
LT417	NA1000 Δ*gcrB* Δ*gcrA*::Ω p*ftsN*::Tn7 (2236602$^+$)	This work
LT418	NA1000 Δ*gcrB* Δ*gcrA*::Ω p*ftsN*::Tn8 (2236750$^+$)	This work
LT419	NA1000 Δ*gcrB* Δ*gcrA*::Ω *ccrM*::Tn (399278$^+$)	This work
LT420	NA1000 *ccrM*::Tn (399278$^+$)	This work
LS2144	NA1000 Δ*ccrM*::Ω p*lacZ*290-PxylX::*ccrM*	[4]
UG2212	NA1000 Δ*ccrM*::Ω	[5]
TPA2357	NA1000 Δ*fljx6* (Δ*flkJKLMNO*)	[6]
LS3118	NA1000 Δ*pilA*	[7]
LT421	NA1000 *rsaA*::pNPTS138	This work
MT65	NA1000 Δ*ftsN* PxylX::*ftsN*	M. Thanbichler
MT237	NA1000 *spmX-mCherry*	[8]
LT491	NA1000 *spmX-mCherry* Δ*gcrA*::Ω	This work
LT492	NA1000 *spmX-mCherry* Δ*gcrA*::Ω *ccrM*::Tn (399278$^+$)	This work
MT174	NA1000 *egfp-parB*	[9]
LT493	NA1000 *egfp-parB* Δ*gcrA*::Ω	This work
LT494	NA1000 *egfp-parB* Δ*gcrA*::Ω *ccrM*::Tn (399278$^+$)	This work

$^+$ *himar1* Tn-insertion location on the NA1000 *Caulobacter crescentus* chromosome.

Plasmids

	Characteristics	Reference/source
pHPV414	non replicative vector in *C. crescentus* harboring the *himar1* transposon (Kan^R)	[10]
pNPTS138	non replicative vector in *C. crescentus* containing the *sacB* gene (Kan^R)	D. Alley, unpublished
pNPTS138-Δ*gcrB*-KO	pNPTS138 allowing *gcrB* deletion (double recombination, sucrose counter selection)	This work
pNPTS138-Δ*rsaA*	pNPTS138 allowing *rsaA* disruption (plasmid integration)	This work
pMT335	High copy number vector containing the leaky P*van* inducible promoter ($Gent^R$)	[11]
pMT335*gcrA*	pMT335 containing *gcrA* coding sequence	This work
pMT335*gcrB*	pMT335 containing *gcrB* coding sequence	This work
pMT335*ftsN*	pMT335 containing *ftsN* coding sequence	This work
pMT335-*RBS-ccrM*	pMT335 containing *ccrM* coding sequence under an optimized RBS	This work
P*lacZ*290	Low copy number vector containing *lacZ* gene (used to create *lacZ* transcriptional fusion) (Tet^R)	[12]
P*lacZ*290-p*ftsN*	P*lacZ*290 containing *ftsN* promoter region (-469 to +228 relative to the ATG)	This work
P*lacZ*290-p*ftsN**	P*lacZ*290-p*ftsN* harboring the A→T mutation in position -52 relative to the ATG (G<u>A</u>TTC→G<u>T</u>TTC)	This work

Oligonucleotides

Names	Sequences
PxylX	5'-AGGATTTCGCGCTGGTCAGACAA-3'
gcrA-EcoRI	5'-AAAAAAGAATTCTTAGATGTAGCGGCGAAGCGA-3'
pro-gcrA	5'-AAAAAACATATGACGGGCGTGGGGACGCCCTCA-3'
gcrB-NdeI	5'-AAAAAACATATGGACTGGAGCGAAGAACGGA-3'
gcrB-EcoRI	5'-AAAAAAGAATTCAGACCAGCCGGGGCTGGAT-3'
ftsN-NdeI	5'-TTTTGGAGCCTAGCTCATATGTCCGATCCGCA-3'
ftsN-EcoRI	5'-AAAAAAGAATTCACTTTACGAAGCAGGATTTG-3'
ccrM-RBS-EcoRI	5'-AGAATTCAGGAGGTAAAAAAATGAAGTTCGGGCCGGAAACCA-3'
ccrM-XbaI	5'-AAAAAATCTAGAATCAGTTCATCCCCGCCCGCA-3'
delrsaA-EcoRI	5'-AAAAGAATTCACCACGGCCGTGACGGTCACCCAAA-3'
delrsaA-HindIII	5'-AAAAAAGCTTCCGTTGACGTTGGCCACCAGA-3'
delgcrB_1-EcoRI	5'-AAAAAAGAATTCTTCACAGCGTCGGAGAA-3'
delgcrB_1-BamHI	5'-AAAAAAGGATTCTTCGCTCCAGTCCATGATCTT-3'
delgcrB_2-BamHI	5'-AAAAAAGGATTCGTCTGACGCCTTCCAAGCCTAT-3'
delgcrB_2-HindIII	5'-AAAAAAAAGCTTTTGGCCAGACGCGTCATCGACA-3'
pftsN-EcoRI	5'-AAAAAAGAATTCGTGGCCTTCTCGTCGGACAT-3'
pftsN-XbaI	5'-AAAAAATCTAGACGTCTTCATCTGAGCGACTT-3'
Cori-fwd	5'-CGCGGAACGACCCACAAACT-3'
Cori-rev	5'-CAGCCGACCGACCAGAGCA-3'
Ter-fwd	5'-CCGTACGCGACAGGGTGAAATAG-3'
Ter-rev	5'-GACGCGGCGGGCAACAT-3'
himar-Seq2	5'-GATATTGCTGAAGAGCTTGGCGGCGAA-3'
himar-TnSeq	5'-AGACCGGGGACTTATCAGCCAACCTGTTA-3'

Simulation of the Minimal *Caulobacter* Cell Cycle Model

The model contains four varying protein concentrations, [GcrA], [CtrA], [CtrA~P] and [CckA~P]. In constructing the model, we focus on the major processes controlling CtrA dynamics: synthesis, phosphorylation, dephosphorylation and degradation. The differential equations describing the model are presented in Figure 1C of the main text.

We do not explicitly include DnaA (or its activity regulator HdaA) due to its primary role being in controlling the frequency of DNA replication rather than asymmetric replication competence [13,14,15]. Instead we take a low threshold in CtrA~P levels (CtrA~P_{min}) as being synchronous with replication initiation. This assumption is consistent with the observed accumulation of DnaA and CtrA [16,17] and their binding to *Cori* [18], see main text. In addition, the regulation of DnaA is still unclear: as stated in the main text, methylation does not significantly alter *dnaA* promoter activity, leaving open the question of what causes the burst in DnaA synthesis prior to replication initiation.

Like most histidine kinases, the membrane-bound protein CckA is bi-functional and when not stimulated as a kinase by DivL, it effects the reversal of the CtrA phosphorylation and stabilisation pathway [19]. However, it has also been shown that dephosphorylation via the reverse phosphorelay is not essential, likely due to the presence of other phosphotases [19]. Furthermore, the stalked pole localisation of CckA, where its stimulation by DivL is blocked [20], is not as consistent as at the swarmer pole [21], implying that this reverse pathway may not always be active. As a result, we do not explicitly include CtrA dephosphorylation via this pathway in our model, though we do include a generic first order dephosphorylation term. It has been suggested [22] that a CtrA~P gradient may form prior to compartmentalisation so that replicative asymmetry is enforced earlier in the cycle and that this pre-existing asymmetry is then locked in on compartmentalisation. Although such a pre-compartmentalisation gradient may form, it is likely not a strict requirement since an

asymmetry in CtrA~P levels will inevitably result after compartmentalisation. We therefore do not include a CtrA~P gradient in our minimal model.

GcrA production is repressed by CtrA~P and this is modelled using a Michaelis-Menten function (Equation 1 of Figure 1C). The proteolysis of GcrA is cell cycle regulated [16] and we incorporate this by employing three different degradation rates: one in the swarmer compartment/cell, one in the stalked compartment and early stalked cell and an intermediate rate in predivisional cells from the time of *ctrA* P_1 hemi-methylation to the time of compartmentalisation (Equation 1). This latter rate is based on the assumption that the regulated proteolysis of GcrA in the swarmer cell/compartment is partially active prior to compartmentalisation.

The total CckA concentration is constant [23,24] so that we need only have an equation for [CckA~P] (Equation 4). The phosphorylation and dephosphorylation reactions are modelled using first order kinetics, with the concentration of CckA~P also being dependent on the value of a phenomenological parameter S as described below. In predivisional cells, CckA phosphorylation is promoted by co-localisation at the nascent swarmer pole with membrane-bound PleC [20]. The polar concentration of PleC is assumed to increase in stalked/predivisional cells in accordance with the approximate doubling in the total PleC concentration [25]. We assume that the resulting increase in the rate of CckA phosphorylation at the swarmer pole compensates for the dilution of the approximately fixed area of the polar region relative to the total membrane area as the cell size increases and we therefore do not incorporate either effect into the first term of Equation 4. Furthermore, the reduced rate of CckA phosphorylation due to the abrupt delocalisation of CckA (and most likely DivL [26]) sometime between compartmentalisation and cell division [21] is assumed to be compensated by the doubling of the polar to total membrane area ratio due to cell division, consistent with maintained CckA and CtrA phosphorylation in swarmer cells [27]. The phosphorelay initiated by CckA~P is not modelled explicitly but instead we implement phosphorylation of CtrA by CckA~P directly using second order kinetics i.e. we take CckA~P

as a proxy for the phosphorylated phosphotransferase ChpT~P. Likewise, unphosphorylated CckA is taken as the representative protein promoting CtrA and CtrA~P proteolysis in second order reactions with the same reaction rate constant (Equations 2, 3). Dephosphorylation of CtrA~P is implemented using first order kinetics (Equations 2, 3).

After proteolysis has reduced [CtrA~P] to less than CtrA~P_{min}, replication initiation is assumed to have occurred. A fixed time (T_R) later, S is switched from 0 to 1 as a signal that the replication fork has moved past the *ctrA* P_1 promoter. Meanwhile, the level of GcrA has risen and, along with the hemi-methylation of *ctrA* P_1, activates CtrA synthesis. This replication initiation dependent event is modelled by a factor of S in the first CtrA synthesis term, representing transcription from the *ctrA* P_1 promoter (Equation 2). A constant parameter, m, implements the lower rate of transcription of the fully methylated *ctrA* P_1 promoter [28]. This term also contains a factor implementing negative feedback from CtrA~P. The second CtrA synthesis term is due to production from the *ctrA* P_2 promoter and contains a factor describing the positive feedback from CtrA~P. In all cases, transcriptional regulation by CtrA~P is modelled, in the absence of data to the contrary, by Michaelis-Menten functions with Hill co-efficients of unity.

Replication initiation is a necessary checkpoint for CckA activation and localisation [29] and indeed CckA~P levels begin to rise shortly after replication initiation has occurred [27]. CckA is observed to be strongly localised to the nascent swarmer pole from about 70 min into the cycle (after synchronisation) and until sometime after cytokinesis but before cell separation [21]. For the sake of simplicity, in our minimal model we increase the rate of CckA phosphorylation when S is switched from 0 to 1, i.e. at the same time as *ctrA* P_1 becomes hemi-methylated (Equation 4). As CckA~P levels rise, they promote CtrA phosphorylation and reduce the proteolysis of CtrA and CtrA~P.

Compartmentalisation is assumed to occur at or shortly before CtrA levels reach their maximum, which is consistent with existing data [3,30] and the fact that high levels of CtrA

are required to activate the late cell division operon, *ftsQA* [31]. In the stalked compartment this event switches S from 1 to 0, where we take [CtrA~P] rising to more than CtrA~P_{max} as the switching threshold. This in turn causes CckA~P levels to decrease exponentially in the stalked compartment mimicking the isolation of that compartment from DivL, the activator of CckA. The CckA~P concentration in the stalked compartment falls and proteolysis of CtrA and CtrA~P increases.

Re-methylation of the *ctrA* P_1 promoter occurs late in the cell cycle and we take it to be synchronous with compartmentalisation. This is implemented in the model by the same factor of S as mentioned above. Since the P_1 promoter is repressed by high CtrA~P and low GcrA levels, when exactly in the late predivisional cell P_1 methylation occurs is not important from the point of view of the mathematical model.

As the levels of CtrA drop in the stalked compartment/cell, the *ctrA* P_1 promoter remains inactive due its full methylation and lack of GcrA. Meanwhile, in the swarmer compartment/cell, CtrA levels remain high and S is only switched from 1 to 0 some time (T_{SW}) after CtrA~P levels exceed CtrA~P_{max}, corresponding to the unknown signals initiating swarmer to stalked differentiation. For the sake of simplicity, re-methylation of *ctrA* P_1 is also taken to occur at this time. For the same reasons as given above for the stalked compartment, when exactly P_1 is re-methylated between the time of compartmentalisation and the initiation of the swarmer to stalked transition does not affect the outcome of the model. The table of model parameters describes the parameters of the model and justification of their values. Further justification for some parameters is given below.

The system of ordinary differential equations (ODE) constituting the model (Figure 1C) was solved using the ode45 solver of MATLAB. Note that the swarmer and stalked cycles were computed separately, with protein concentrations tracked into the appropriate compartment on compartmentalisation. Profiles of the GcrA and total CtrA concentrations in the swarmer cell cycle were calculated and plotted once their oscillations became periodic (Figure S1A).

Hence the profiles are not dependent on the particular initial conditions chosen to solve the ODE system. The protein values shortly after the initiation of the swarmer to stalked transition were then used as the initial conditions for separately solving the ODE system of the stalked cell cycle (Figure S1B). The first stalked cell cycle is distinct from subsequent cycles since there is no inheritance of certain cell-type specific proteins from the previous cycle. Experimentally, for example, GcrA is undetectable in swarmer cells [3,16] so that it must be synthesised at the beginning of the first stalked cycle, whereas in the following stalked cycles it can be inherited. In order to make the profiles comparable with immunoblot data of imperfectly synchronised cultures (a synchronised culture is started by isolating swarmer cells from a mixed population), we averaged over 800 swarmer cell cycle profiles that were out of phase with each other by at most 30 min. The phases were drawn from a truncated normal distribution drawn over the interval [-15 min, 15 min] with a mean of 0 and an underlying standard deviation of 5 min. At the end of the cycle, 800 swarmer cells have become 800 swarmer and 800 stalked cells resulting in loss of periodicity in the overall CtrA and GcrA profiles. Hence, one cycle of these convolved, swarmer cell cycle profiles were then averaged with their corresponding convolved, (first) stalked cell cycle profiles with a weighting corresponding to the compartment volumes (see below) and plotted (Figure 2B and Figure S2A). These profiles were compared to quantified protein immunoblots of GcrA and CtrA taken from a synchronised culture over the duration of one swarmer cell cycle and the best fit was found by choosing parameters by hand (see table). A parameter fitting algorithm based on simulated annealing was also employed but did not lead to a better fit. There are two factors that we believe contribute to the poorer fit of the last few data points, especially for GcrA. The first cell cycle after synchronisation has a longer cell cycle period than subsequent cycles due to the shock of the synchronisation procedure [32]. As we have not incorporated this effect, we therefore expect our model to show longer subsequent cell cycles and therefore, for example, a delay in the re-accumulation of GcrA at beginning of the second stalked cell cycle, which would contribute to the off-set in GcrA levels seen in Figure

2B. Secondly, the absence of DnaA regulation of *gcrA* transcription might also contribute to the poorer fit of the model to the GcrA immunoblot data.

The model is quite adaptable. For example, by modifying the two time durations T_{SW} and T_{R} and/or the switching of the discrete variable S it is possible to mimic carbon and nitrogen starvation conditions [17]. For example, nitrogen starvation blocks swarmer cell differentiation. This could effectively be modelled by making the time duration T_{SW} arbitrarily long. The same conditions cause stalked cells to arrest as pinched predivisional cells. This could be modelled by raising the threshold CtrA~P$_{\text{max}}$ sufficiently such that compartmentalisation does not occur, maintaining high CtrA levels in both compartments, stalling the cycle.

We also examined the ability of the model to fit previously published GcrA and CtrA data [3]. Good fits were straightforwardly obtained in all cases (data not shown).

CckA

In early stalked cells, the concentration of CckA~P is about 30% of its predivisional maximum [27]. In our fitting, we have that at this maximum, in predivisional cells, 90% of CckA is phosphorylated. We can therefore use a steady state approximation to get estimates for two model parameters:

$$\frac{[\text{CckA\textasciitilde P}]}{\text{CckA}_T} \approx \frac{k_{p,bk,\text{CckA}} + k_{p,\text{CckA}}}{k_{p,bk,\text{CckA}} + k_{p,\text{CckA}} + k_{dp,\text{CckA\textasciitilde P}}} = 0.9 \quad \text{in predivisional cells,}$$

$$\frac{[\text{CckA\textasciitilde P}]}{\text{CckA}_T} \approx \frac{k_{p,bk,\text{CckA}}}{k_{p,bk,\text{CckA}} + k_{dp,\text{CckA\textasciitilde P}}} = 0.3 \times 0.9 \quad \text{in early stalked cells.}$$

We then find

$$\frac{k_{p,bk,\text{CckA}}}{k_{dp,\text{CckA\textasciitilde P}}} = 0.37, \qquad \frac{k_{p,\text{CckA}}}{k_{dp,\text{CckA\textasciitilde P}}} = 8.6.$$

This approximation is valid as long as the timescale for CckA dephosphorylation is sufficiently short with respect to the length of the cell cycle. [CckA~P] can then reach its steady state level before the next switching of S occurs and be consistent with the aforementioned experimental result. The model can be easily modified as quantitative experimental data becomes available for CckA expression.

CtrA Concentration

We take a mixed population average cell volume of 0.42 μm^3 based on an average cell length of 3.15 μm [21] and average cell width of 0.43 μm [33]. Published length and width ratios of the two compartments in predivisional cells [34] suggest a swarmer to stalked compartment volume ratio of approximately 44:56. Using this result and the volume distribution given in [35], we deduce that the average cell volume is about 72% of the predivisional cell volume. Hence, we take a predivisional cell volume of 0.58 μm^3. Given 18000 molecules of CtrA in the late predivisional cell [36], this implies a total CtrA concentration in late predivisional cells of 51 μM. In the early stalked cell the total CtrA concentration drops to around 2% of its predivisional maximum [3,16,37,38], consistent with our experimental findings (Figure 2B), which implies a minimum total CtrA concentration of about 1 μM (~200 molecules).

CtrA Phosphorylation

Using existing data in the literature it is possible to quantitatively examine the phosphorylation levels of CtrA and constrain several parameters of the model. Semi-quantitative immunoblots showing total CtrA concentrations, normalized by their maximum value, throughout the cell cycle have been given by several authors [3,16,37,38] as well as in the present report (Figure 2B). The number of CtrA~P molecules, normalized by its maximum value, has also been quantified during the cell cycle [27], and we convert this into a normalized concentration. By recognising that at each point in the cell cycle the (unnormalized) CtrA~P concentration must be less than the total (unnormalized) CtrA

concentration, comparing the two above normalized data sets gives an upper bound for the fraction of phosphorylated CtrA in the predivisional cell. The phosphorylation percentage peaks, at about 15 times the late predivisional value, at an earlier point in the cell cycle approximately 20 min before CtrA levels begin to increase. If CtrA is 100% phosphorylated at this point in the early stalked cell, the phosphorylation level in predivisional cells would be around 7%, which is much lower than in previous cell cycle models. One explanation for this novel observation is that the phosphorylation reaction is substrate saturating, which is consistent with the known number of CtrA molecules in the predivisional cell (18,000-22,000 molecules [30,36]) and the much lower levels of CckA and the phosphotransferase ChpT [19]. Hence, while the concentration of CtrA~P rises, the percentage of CtrA that is phosphorylated actually decreases. Due to the additional parameters required to model such phosphorylation kinetics and the lack of direct experimental evidence, we have not incorporated saturation of the phosphorylation reaction into our minimal model. However, we do employ 7% CtrA phosphorylation in predivisional cells as a constraint in our parameter fitting. Since CtrA phosphorylation reactions occur on a much faster timescale (of the order of seconds or less [22]) compared to that of changes in the CtrA concentration, and our parameter choices are consistent with this, we can use a steady state approximation for CtrA~P levels

$$\frac{[\text{CtrA\textasciitilde P}]}{[\text{CtrA}]+[\text{CtrA\textasciitilde P}]} \approx \frac{k_{p,\text{CtrA}}[\text{CckA\textasciitilde P}]}{k_{p,\text{CtrA}}[\text{CckA\textasciitilde P}] + k_{dp,\text{CtrA\textasciitilde P}}}.$$

By setting this ratio to be 0.07 in predivisional cells and using the predivisional steady state approximation for CckA~P levels (see above), we can constrain the ratio between the phosphorylation and dephosphorylation rates for CtrA.

Given the above estimate of 51 μM for the total CtrA concentration level in predivisional cells, this implies a predivisional CtrA~P concentration of about 3500 nM and we take this to be the switching threshold CtrA~P_{max}. The lower switching threshold CtrA~P_{min} is chosen for the best fit to the GcrA and CtrA immunoblot data.

Testing the Model

To test the model, we investigated the behaviour of the *ctrA* P_1 promoter in various simulated mutants and compared to experiments. P_1 is believed to be repressed when it is fully methylated and active when it is unmethylated or hemi-methylated [28]. We found *in silico* that maintaining *ctrA* P_1 in its hemi-methylated state only slightly decreased the cell cycle period (Figure S2B). This result is consistent with the minor change in cell cycle timing observed in the WT compared to a strain without P_1 methylation, where plasmid-expressed *ctrA* is under the control of a mutant promoter with the P_1 methylation site eliminated by point mutation [28]. We also examined the effect in the model of keeping the P_1 promoter fully methylated. As expected, *ctrA* P_1 transcription was severely repressed and the cell cycle period was lengthened (by about 10%). This effect is phenocopied by placing *ctrA* near the chromosome terminus, a location that remains fully methylated throughout most of the cell cycle [28]. Indeed, maintaining chromosome-wide full methylation by constitutively overexpressing CcrM is non-lethal [39]. Recently, *ctrA* P_1 was disrupted by a different means - the insertion of a 5 bp spacer and cells with this insertion were again found to have a lengthened cell cycle [40].

We next mimicked constitutive overexpression of GcrA by increasing its rate of synthesis *in silico* by a factor of two. This had little effect on the predicted profiles of CtrA and shortened the cell cycle period by only about 6%. These results are consistent with prior work showing the near WT phenotype of a *gcrA* overexpression strain [3,16]. GcrA levels still vary in this mutant due to regulated proteolysis [16], an effect included in our model. However, *in silico*, we can test the effect of constant GcrA levels. We again predict only a small decrease of cell cycle time (8%) due to the presence of two other regulators of P_1 transcription: methylation and CtrA~P. We also tested the effect of maintaining full P_1 methylation as above when GcrA is overexpressed. In this case, we predict that the cell cycle period is less drastically affected than in the case of full P_1 methylation alone, with now only a 6% increase in period over the

WT. This is due to the resulting small increase in the low level of GcrA-promoted transcription from the fully methylated P_1 promoter.

Comparison with Existing *Caulobacter* Cell Cycle Models

Our approach has utilised a hybrid model, containing both continuous differential equations and discrete switches and, in this respect, is similar to that of Shen et al. [41]. However, we have focused on minimal modelling and have endeavoured to include only clearly justifiable discrete events, switches and time durations. Such discreteness reduces the complexity of a fully continuous model, such as that seen in the models of Li et al. [42,43], while maintaining biological relevance. Indeed, Lin et al. have used a somewhat similar approach in constructing a simplified model of the stalked cell cycle only, with a focus on *divJ*-dependent regulation of noise in the cell cycle period [44]. However that model did not incorporate dependencies on key cell cycle events, e.g. both the re-accumulation and activation of CtrA in the early stalked cell are dependent on DNA replication initiation having occurred, which is itself contingent on low CtrA~P levels. In their model, the CckA phosphorelay is controlled in such a way that its re-activation can occur without CtrA~P levels first being substantially reduced, allowing the next cell cycle to begin without replication initiation actually occurring. Finally, in our analysis we have also incorporated recent results on the regulation of the cell cycle [13,18,20].

Model Parameters

Symbol	Description	Value	Justification
$k_{s,\text{GcrA}}$	Maximum synthesis rate of GcrA	0.5 (nM s^{-1})	Value does not affect model outcome after normalisation.
J_{CG}	Binding constant between CtrA~P and *gcrA* promoter	155 (nM)	Chosen by best fit of convolved, compartment averaged, normalised GcrA profile to normalised immunoblot data. GcrA levels begin to drop as soon as CtrA accumulates suggesting a low binding constant relative to maximum CtrA~P levels. See text.
$k^{\text{SW}}{}_{d,\text{GcrA}}$	Degradation rate of GcrA in swarmer cells	$\frac{\ln 2}{60 \times 10.5}$ (s^{-1})	Half-life of GcrA in swarmer cells is 10.5 min [16].
$k^{\text{ST}}{}_{d,\text{GcrA}}$	Degradation rate of GcrA in stalked cells	$\frac{\ln 2}{60 \times 42}$ (s^{-1})	Half-life of GcrA in stalked cells is 42 min [16].
$k^{\text{PD}}{}_{d,\text{GcrA}}$	Degradation rate of GcrA in predivisional cells	$\frac{\ln 2}{60 \times 21}$ (s^{-1})	Chosen for best fit to data. See text.
$k_{s1,bk,\text{CtrA}}$	Basal production rate of CtrA from P_1 promoter	3.2 (nM s^{-1})	The P_1 promoter maintains activity after GcrA depletion [3]
$k_{s1,\text{CtrA}}$	Production rate of CtrA from P_1 promoter due to GcrA per nM of GcrA	0.026 (s^{-1})	Chosen for best fit to data and so that synthesis from *ctrA* promoters qualitatively matches published promoter expression profiles [28,36,45].
$k_{s2,\text{CtrA}}$	Maximum synthesis rate of CtrA from P_2 promoter	32 (nM s^{-1})	
J_{C1}	Dissociation constant between CtrA~P and *ctrA* P_1 promoter	400 (nM)	Chosen so that synthesis from *ctrA* promoters qualitatively matches published promoter expression profiles [28,36,45] and to be consistent with estimates of CtrA~P levels. See text.
J_{C2}	Dissociation constant between CtrA~P and *ctrA* P_2 promoter	900 (nM)	
m	Ratio of transcription rates between fully methylated and hemi-methylated *ctrA* P_1 promoter.	0.15	Full methylation of P_1 strongly represses transcription [28].
$k_{dp,\text{CtrA\textasciitilde P}}$	Reaction rate constant for CtrA~P dephosphorylation	$\frac{\ln 2}{5}$(s^{-1})	The half-life for CtrA~P ≤ 8 s [22]. Since it is much faster than the timescale for changes in total CtrA concentration, the exact value of this parameter does not affect model outcome.
$k_{p,\text{CtrA}}$	Reaction rate constant for CtrA~P phosphorylation	$\frac{7}{93}\frac{k_{dp,\text{CtrA\textasciitilde P}}}{0.9 \times \text{CckA}_T}$ (nM^{-1} s^{-1})	Phosphorylation rate constant is chosen to

			achieve 7% CtrA phosphorylation in predivisional cells when CckA~P levels are at their maximum. See text.
$k_{d,\text{CtrA}}$	Reaction rate constant for CtrA and CtrA~P degradation	$\frac{\ln 2}{60\times 5\times(1-0.3\times 0.9)\times \text{CckA}_T}$ (nM^{-1} s^{-1})	The half-life of CtrA is as short as 5 min (during the SW-ST transition) [46]. With the value shown the half-life varies from 5 min to 37 min during the course of the cycle according to CckA levels.
	Maximum percentage of CckA phosphorylation	90% of total CckA	Chosen for best fit to data. See text.
$k_{dp,\text{CckA\~P}}$	Reaction rate constant for CckA~P dephosphorylation	$\frac{\ln 2}{60\times 5}$ (s^{-1})	See text. The precise value does not significantly affect the model output.
$k_{p,bk,\text{CckA}}$	Reaction rate constant for CckA background phosphorylation	$0.37\times k_{dp,\text{CckA\~P}}$ (s^{-1})	See text.
$k_{p,\text{CckA}}$	Reaction rate constant for CckA phosphorylation due to DivL	$8.6\times k_{dp,\text{CckA\~P}}$ (s^{-1})	See text.
CckA_T	Total concentration of CckA	150 (nM)	Estimate of 55 molecules in predivisional cells [22]. This parameter can be normalised out.
CtrA~P_{min}	Threshold of CtrA~P levels to allow replication initiation	150 (nM)	Chosen together with T_{R} for best fit to data. See text.
CtrA~P_{max}	Threshold of CtrA~P levels taken as coincident with compartmentalisation	3500 (nM)	Assuming 7% CtrA phosphorylation. Total CtrA peaks at around 50 μM. See text.
T_{SW}	Time from compartmentalisation to initiation of swarmer to stalked differentiation of daughter swarmer cell	36 (min)	Based on best fit of model to immunoblot data.
T_{R}	Time from CtrA~P dropping below CtrA~P_{min} to the hemi-methylation of the *ctrA* P_1 promoter	57 (min)	Chosen together with CtrA~P_{min} for best fit of model to immunoblot data.

References

1. Simon R, Priefer U, Puhler A (1983) A Broad Host Range Mobilization System for In Vivo Genetic Engineering: Transposon Mutagenesis in Gram Negative Bacteria. Nat Biotech 1: 784-791.
2. Evinger M, Agabian N (1977) Envelope-associated nucleoid from *Caulobacter crescentus* stalked and swarmer cells. J Bacteriol 132: 294-301.
3. Holtzendorff J, Hung D, Brende P, Reisenauer A, Viollier PH, et al. (2004) Oscillating global regulators control the genetic circuit driving a bacterial cell cycle. Science 304: 983-987.
4. Stephens C, Reisenauer A, Wright R, Shapiro L (1996) A cell cycle-regulated bacterial DNA methyltransferase is essential for viability. Proc Natl Acad Sci U S A 93: 1210-1214.
5. Fioravanti A, Fumeaux C, Mohapatra SS, Bompard C, Brilli M, et al. (2013) DNA Binding of the Cell Cycle Transcriptional Regulator GcrA Depends on N6-Adenosine Methylation in *Caulobacter crescentus* and Other *Alphaproteobacteria*. PLoS Genet 9: e1003541.
6. Faulds-Pain A, Birchall C, Aldridge C, Smith WD, Grimaldi G, et al. (2011) Flagellin redundancy in *Caulobacter crescentus* and its implications for flagellar filament assembly. J Bacteriol 193: 2695-2707.
7. Skerker JM, Shapiro L (2000) Identification and cell cycle control of a novel pilus system in *Caulobacter crescentus*. Embo J 19: 3223-3234.
8. Radhakrishnan SK, Thanbichler M, Viollier PH (2008) The dynamic interplay between a cell fate determinant and a lysozyme homolog drives the asymmetric division cycle of *Caulobacter crescentus*. Genes Dev 22: 212-225.
9. Thanbichler M, Shapiro L (2006) MipZ, a spatial regulator coordinating chromosome segregation with cell division in *Caulobacter*. Cell 126: 147-162.
10. Viollier PH, Thanbichler M, McGrath PT, West L, Meewan M, et al. (2004) Rapid and sequential movement of individual chromosomal loci to specific subcellular locations during bacterial DNA replication. Proc Natl Acad Sci U S A 101: 9257-9262.
11. Thanbichler M, Iniesta AA, Shapiro L (2007) A comprehensive set of plasmids for vanillate- and xylose-inducible gene expression in *Caulobacter crescentus*. Nucleic Acids Res 35: e137.
12. Gober JW, Shapiro L (1992) A developmentally regulated *Caulobacter* flagellar promoter is activated by 3' enhancer and IHF binding elements. Mol Biol Cell 3: 913-926.
13. Jonas K, Chen YE, Laub MT (2011) Modularity of the bacterial cell cycle enables independent spatial and temporal control of DNA replication. Curr Biol 21: 1092-1101.
14. Fernandez-Fernandez C, Gonzalez D, Collier J (2011) Regulation of the activity of the dual-function DnaA protein in *Caulobacter crescentus*. PLoS One 6: e26028.
15. Collier J, Shapiro L (2009) Feedback control of DnaA-mediated replication initiation by replisome-associated HdaA protein in *Caulobacter*. J Bacteriol 191: 5706-5716.
16. Collier J, Murray SR, Shapiro L (2006) DnaA couples DNA replication and the expression of two cell cycle master regulators. Embo J 25: 346-356.
17. Gorbatyuk B, Marczynski GT (2005) Regulated degradation of chromosome replication proteins DnaA and CtrA in *Caulobacter crescentus*. Mol Microbiol 55: 1233-1245.
18. Taylor JA, Ouimet MC, Wargachuk R, Marczynski GT (2011) The *Caulobacter crescentus* chromosome replication origin evolved two classes of weak DnaA binding sites. Mol Microbiol 82: 312-326.
19. Chen YE, Tsokos CG, Biondi EG, Perchuk BS, Laub MT (2009) Dynamics of Two Phosphorelays Controlling Cell Cycle Progression in *Caulobacter crescentus*. J Bacteriol 191: 7417-7429.
20. Tsokos CG, Perchuk BS, Laub MT (2011) A dynamic complex of signaling proteins uses polar localization to regulate cell-fate asymmetry in *Caulobacter crescentus*. Dev Cell 20: 329-341.

21. Angelastro PS, Sliusarenko O, Jacobs-Wagner C (2010) Polar localization of the CckA histidine kinase and cell cycle periodicity of the essential master regulator CtrA in *Caulobacter crescentus*. J Bacteriol 192: 539-552.
22. Chen YE, Tropini C, Jonas K, Tsokos CG, Huang KC, et al. (2011) Spatial gradient of protein phosphorylation underlies replicative asymmetry in a bacterium. Proc Natl Acad Sci U S A 108: 1052-1057.
23. Tan MH, Kozdon JB, Shen X, Shapiro L, McAdams HH (2010) An essential transcription factor, SciP, enhances robustness of *Caulobacter* cell cycle regulation. Proc Natl Acad Sci U S A 107: 18985-18990.
24. Jacobs C, Domian IJ, Maddock JR, Shapiro L (1999) Cell cycle-dependent polar localization of an essential bacterial histidine kinase that controls DNA replication and cell division. Cell 97: 111-120.
25. Viollier PH, Sternheim N, Shapiro L (2002) A dynamically localized histidine kinase controls the asymmetric distribution of polar pili proteins. Embo J 21: 4420-4428.
26. Sciochetti SA, Ohta N, Newton A (2005) The role of polar localization in the function of an essential *Caulobacter crescentus* tyrosine kinase. Mol Microbiol 56: 1467-1480.
27. Jacobs C, Ausmees N, Cordwell SJ, Shapiro L, Laub MT (2003) Functions of the CckA histidine kinase in *Caulobacter* cell cycle control. Mol Microbiol 47: 1279-1290.
28. Reisenauer A, Shapiro L (2002) DNA methylation affects the cell cycle transcription of the CtrA global regulator in *Caulobacter*. Embo J 21: 4969-4977.
29. Iniesta AA, Hillson NJ, Shapiro L (2010) Polar remodeling and histidine kinase activation, which is essential for *Caulobacter* cell cycle progression, are dependent on DNA replication initiation. J Bacteriol 192: 3893-3902.
30. Judd EM, Ryan KR, Moerner WE, Shapiro L, McAdams HH (2003) Fluorescence bleaching reveals asymmetric compartment formation prior to cell division in *Caulobacter*. Proc Natl Acad Sci U S A 100: 8235-8240.
31. Wortinger M, Sackett MJ, Brun YV (2000) CtrA mediates a DNA replication checkpoint that prevents cell division in *Caulobacter crescentus*. Embo J 19: 4503-4512.
32. Poindexter JS (1964) Biological Properties and Classification of the *Caulobacter* Group. Bacteriol Rev 28: 231-295.
33. Fukuda A, Iba H, Okada Y (1977) Stalkless mutants of *Caulobacter crescentus*. J Bacteriol 131: 280-287.
34. Sliusarenko O, Heinritz J, Emonet T, Jacobs-Wagner C (2011) High-throughput, subpixel precision analysis of bacterial morphogenesis and intracellular spatio-temporal dynamics. Mol Microbiol 80: 612-627.
35. Siegal-Gaskins D, Ash JN, Crosson S (2009) Model-based deconvolution of cell cycle time-series data reveals gene expression details at high resolution. PLoS computational biology 5: e1000460.
36. Spencer W, Siam R, Ouimet MC, Bastedo DP, Marczynski GT (2009) CtrA, a global response regulator, uses a distinct second category of weak DNA binding sites for cell cycle transcription control in *Caulobacter crescentus*. J Bacteriol 191: 5458-5470.
37. McGrath PT, Iniesta AA, Ryan KR, Shapiro L, McAdams HH (2006) A dynamically localized protease complex and a polar specificity factor control a cell cycle master regulator. Cell 124: 535-547.
38. Iniesta AA, Shapiro L (2008) A bacterial control circuit integrates polar localization and proteolysis of key regulatory proteins with a phospho-signaling cascade. Proc Natl Acad Sci U S A 105: 16602-16607.
39. Zweiger G, Marczynski G, Shapiro L (1994) A *Caulobacter* DNA methyltransferase that functions only in the predivisional cell. J Mol Biol 235: 472-485.
40. Schredl AT, Perez Mora YG, Herrera A, Cuajungco MP, Murray SR (2012) The *Caulobacter crescentus ctrA* P1 promoter is essential for the coordination of cell cycle events that prevent the overinitiation of DNA replication. Microbiology 158: 2492-2503.
41. Shen X, Collier J, Dill D, Shapiro L, Horowitz M, et al. (2008) Architecture and inherent robustness of a bacterial cell-cycle control system. Proc Natl Acad Sci USA 105: 11340-11345.

42. Li S, Brazhnik P, Sobral B, Tyson JJ (2008) A quantitative study of the division cycle of *Caulobacter crescentus* stalked cells. PLoS Comput Biol 4: e9.
43. Li S, Brazhnik P, Sobral B, Tyson JJ (2009) Temporal controls of the asymmetric cell division cycle in *Caulobacter crescentus*. PLoS Comput Biol 5: e1000463.
44. Lin Y, Crosson S, Scherer NF (2010) Single-gene tuning of *Caulobacter* cell cycle period and noise, swarming motility, and surface adhesion. Mol Syst Biol 6: 445.
45. Domian IJ, Reisenauer A, Shapiro L (1999) Feedback control of a master bacterial cell-cycle regulator. Proc Natl Acad Sci U S A 96: 6648-6653.
46. Domian IJ, Quon KC, Shapiro L (1997) Cell type-specific phosphorylation and proteolysis of a transcriptional regulator controls the G1-to-S transition in a bacterial cell cycle. Cell 90: 415-424.
